# Life history, climate and biogeography interactively affect worldwide genetic diversity of plant and animal populations

**DOI:** 10.1038/s41467-021-20958-2

**Published:** 2021-01-22

**Authors:** H. De Kort, J. G. Prunier, S. Ducatez, O. Honnay, M. Baguette, V. M. Stevens, S. Blanchet

**Affiliations:** 1 Plant Conservation and Population Biology, Department of Biology, University of Leuven, Heverlee, Belgium; 2 Centre National de la Recherche Scientifique, SETE Station d’Ecologie Théorique et Expérimentale, UMR 5321, Moulis, France; 3 Department of Earth Sciences, University of Cambridge, Cambridge, UK; 4 Institut Systématique, Evolution, Biodiversité (ISYEB), UMR 7205 Museum National d’Histoire Naturelle, CNRS, Sorbonne Université, EPHE, Université des Antilles, Paris, France

**Keywords:** Conservation biology, Evolutionary genetics, Genetics, Plant sciences, Zoology

## Abstract

Understanding how biological and environmental factors interactively shape the global distribution of plant and animal genetic diversity is fundamental to biodiversity conservation. Genetic diversity measured in local populations (GD_P_) is correspondingly assumed representative for population fitness and eco-evolutionary dynamics. For 8356 populations across the globe, we report that plants systematically display much lower GD_P_ than animals, and that life history traits shape GD_P_ patterns both directly (animal longevity and size), and indirectly by mediating core-periphery patterns (animal fecundity and plant dispersal). Particularly in some plant groups, peripheral populations can sustain similar GD_P_ as core populations, emphasizing their potential conservation value. We further find surprisingly weak support for general latitudinal GD_P_ trends. Finally, contemporary rather than past climate contributes to the spatial distribution of GD_P_, suggesting that contemporary environmental changes affect global patterns of GD_P_. Our findings generate new perspectives for the conservation of genetic resources at worldwide and taxonomic-wide scales.

## Abstract (web summary)

A global analysis of population-level variation in genetic diversity for 727 plant and animal species finds that biogeography, life history traits and climate are important for predicting the distribution of local genetic diversity, and should be considered together when assessing the local conservation status of species.

## Introduction

Human-mediated environmental changes disrupt population and community dynamics, resulting in population genetic diversity loss, species extinctions, changes in ecosystem functioning and loss of ecosystem services^1–5^. Through its relation with inbreeding and demographic processes^6,7^, population genetic diversity (GD_P_) is often used to support and complement the identification of “evolutionary significant units” (ESUs, Supplementary Notes 1), upon which conservation programs frequently rely to inform about the evolutionary and demographic history of populations^8,9^. GD_P_ depends on species-specific life-history traits, population dynamics, past climatic and demographic events, biogeography, and local and global environmental factors^10,11^. Despite the importance of local population dynamics in shaping species’ ranges and communities^12–14^, the relative importance of local and range-wide processes in driving GD_P_ remains a major knowledge gap. Moreover, understanding the complex relationships between GD_P_, species traits, biogeography and environmental gradients is key to the establishment of general conservation guidelines that are valid across taxa and space^15–17^.

Several studies have evaluated how GD_**P**_ varies among species’ life-history traits (e.g. effect of lifespan^18^) or with biogeography (e.g. core vs. periphery effects^10^). However, these studies typically focus on one particular driver of GD_P_, or consider them independently, whereas variation in GD_P_ more likely results from interactions between life-history-related, climatic, historical and biogeographic factors. For instance, wind-pollinated plant populations with high outcrossing rates and/or wind-dispersed seeds generally sustain high genetic diversity due to positive effects of these traits on effective population size (N_E_) and rates of molecular evolution^19,20^, yet other studies have questioned the generality of this pattern^19,21^. Similarly, populations of small animals with high fecundity and short longevity, large geographic ranges, long-distance dispersal and/or with generalist lifestyles have often been found to harbour relatively high levels of genetic diversity^22–27^, whereas other studies could not validate these life-history-related GD_P_ patterns^25,28–30^.

From a biogeographic point of view, populations at species’ distribution edges are often characterized by relatively low genetic diversity due to founder effects and low connectivity, whereas core populations generally harbour higher levels of genetic diversity due to increased admixture of lineages with distinct evolutionary and/or demographic trajectories^10,31–33^. Other studies, however, failed to find evidence for this core-periphery hypothesis^34,35^. Furthermore, while insular conditions have long been acknowledged to limit GD_P_ due to founder effects and reduced gene flow^36–39^, recent work has questioned the generality of this theory^40,41^.

Finally, environmental—and in particular climatic—gradients are also thought to affect GD_P_. Like species diversity, GD_P_ is expected to be higher around the Equator because (i) climatic conditions have been much more stable in the last 10–20,000 years with no major glaciations that could otherwise have generated severe demographic bottlenecks, and (ii) the higher temperatures around the tropics may boost mutation rates and hence GD_p_^11,42,43^ (but see ref. ^44^). Latitudinal clines in GD_P_ have been informed for some species^30,45,46^, but the exact mechanisms sustaining this pattern are still poorly understood and it remains unknown to what extent temperature and precipitation contribute to spatial patterns of GD_P_. Together, the marked heterogeneity in GD_P_ patterns across taxa and space urges for a better global understanding of how species traits, biogeography and climate interactively mediate variation in GD_P_ at a large spatial scale.

While studies examining the combined effects of species traits, biogeography and climate on global levels of GD_P_ are lacking, various quantitative reviews considered both life-history traits and spatial factors as drivers of genetic diversity across populations, i.e., at the species level (hereafter “GD_**S**_”, Supplementary Notes 1)^24,44,47–51^. These studies demonstrated that species distributed around the Equator have higher overall genetic diversity (i.e., GD_S_) than species occurring near the poles, and that GD_**S**_ is lower for long-lived or low-fecundity species than for short-lived or high-fecundity species. However, contrary to GD_**P**_ (Supplementary Notes 1)^52,53^, GD_**S**_ is insensitive to local-scale processes ruled for instance by environmental constraints, biogeographic features or anthropogenic stressors. As opposed to GD_**S**_, GD_**P**_ can thus be linked to local community dynamics through its relationship with population fitness, which in turn affects the ability of species to compete and interact with co-occurring species^12,54,55^. Moreover, while neutral GD_**P**_ partially reflects the demographic population history, part of GD_**P**_ arose through hitchhiking with adaptive genetic variants. As a result, local populations harboring low genetic diversity are expected to have a reduced capacity to cope with changing environmental conditions^6,7^, even if they exhibit high levels of GD_**S**_. Thus, although GD_**S**_ assessments provide crucial insights into the regional gene pools and into species’ demographic trajectories, it is poorly informative in terms of contemporary population dynamics and the susceptibility of individual populations to genetic erosion (Supplementary Notes 1). For conservation purposes, it is of upmost importance to study large spatial and taxonomic patterns of GD_**P**_ across life-history, biogeographic and climatic contexts.

**Fig. 1:**
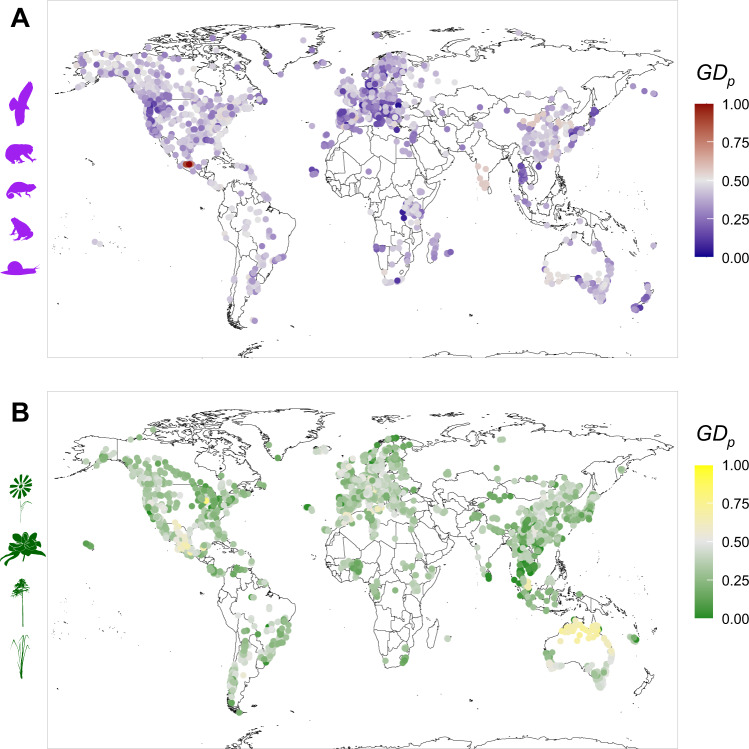
Global distribution of population genetic diversity (GDP) across the animal and the plant kingdoms. Our survey encompasses 8356 local animal (**A**) and plant (**B**) populations throughout the world and for which genetic diversity (measured as multilocus expected heterozygosity) has been assessed. Source data are provided as a Source Data file.

Here, we synthetize published datasets to inform GD_P_ (measured as expected heterozygosity inferred from nuclear markers) patterns across large spatial (worldwide) and taxonomic (across plants and animals) scales, and to identify the main life-history-related, biogeographic and climatic factors sustaining these patterns of GD_P_. Our study involves 8356 populations distributed across the globe and from 242 eudicot, 10 magnolid, 82 monocot, 50 pinopsida (hereafter pines), 51 amphibian, 36 reptile, 44 mollusc, 139 mammal, and 73 bird species (Fig. 1, Tables S1–S3). We quantify relations between life-history traits, biogeography, elevation, past and current climate and GD_P_. In this work, we (i) identify spatial patterns of GD_P_ across phyla and across the globe, and (ii) determine how life-history traits, past and current climatic conditions and biogeography interactively affect GD_P_. Our study provides unique insights into the determinants of GD_P_ and its underlying processes, and improves our general understanding of the drivers of molecular diversity in nature. We discuss the conservation implications of our results and we propose additional endeavours that aim to unravel how various biological and environmental factors impact natural genetic variation.

## Results

### GD_P_ across large taxonomic groups

**Fig. 2:**
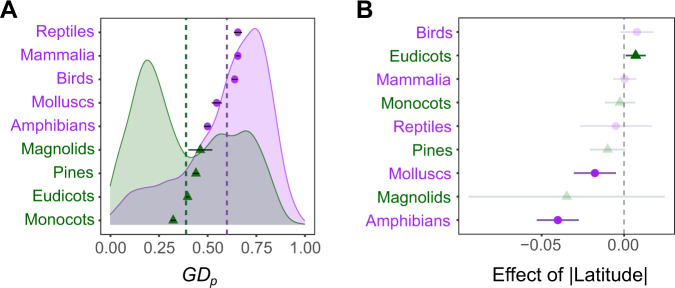
Population genetic diversity (GD_P_) across the plant (green) and animal (purple) kingdom. A density plot (**A**) shows the distribution of raw GD_P_ values in plants and animals, with 95% confidence interval error bars of phylum means. Phylum-specific slopes (with 95% confidence intervals derived from the Phylum model; Supplementary Data 5) of the absolute latitude effect (i.e. distance to equator) show that diversity only decreases away from the equator in amphibians and molluscs, and even increases in eudicot plants (**B**). Non-significant slopes (p-values > 0.05) are transparent. Source data are provided as a Source Data file.

We first explored the global distribution of GD_P_ across the plant and animal kingdoms (Fig. 1). We found a striking difference in GD_P_ between plants and animals, with plants harbouring lower levels of GD_P_ than animals (Figs. 1, 2A), also after controlling for non-independence caused by relatedness and methodological aspects through a linear mixed model (Supplementary Data 4). We further found that GD_P_ significantly decreases away from the equator in animals, but not in plants (Fig. 2B, Supplementary Data 4). However, the latitudinal gradient observed in animals was weak (slope of −0.006 ± 0.002 SE), and heterogeneous across phyla (Fig. 2B, Supplementary Data 5). In particular, mollusc and amphibian species displayed a significant decrease in GD_P_ with increasing distance from the equator, whereas the relationship between GD_P_ and absolute latitude was not significant in other phyla (Fig. 2B). In plants, while there was no overall latitudinal GD_p_ gradient (slope of 0.001 ± 0.002 SE), GD_P_ slightly (but significantly) increased with increasing distance from the equator only in eudicots (Fig. 2B).

### Contribution of climate, life history and biogeography to GD_P_ patterns

To explain spatial and taxonomic variation observed in GD_P_, an exhaustive model was generated for each kingdom separately to assess potential effects of biogeography (i.e. the position of each population relative to the core and edges of the species’ range), life-history traits (longevity, body size and fecundity reduced to two principal components, and species’ range and elevation as proxies for dispersal ability and niche width, see Methods), contemporary climate (temperature, precipitation and humidity) and long-term temperature stability during the Last Glacial Maximum (LGM), and during the mid Holocene (MH) on GD_P_. Together, these fixed effects explained 32.1% (animals) and 10.2% (plants) of global GD_P_ patterns. Models’ residuals did not display signs of spatial autocorrelation, whereas spatial autocorrelation was detected at a very fine spatial scale for the raw GD_P_ data especially for animals (Supplementary Fig. 4). Our models thus adequately dealt with any signs of spatial autocorrelation.

**Fig. 3:**
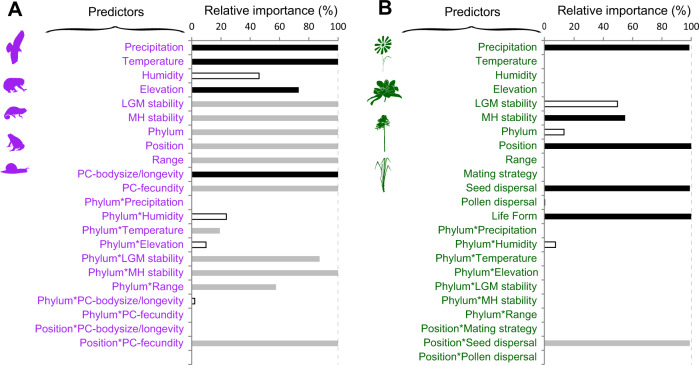
Relative importance of predictors used in the animal and plant kingdom models. An information theoretic approach was used to identify the most important predictors of animal (**A**) and plant (**B**) GD_p_, respectively. Predictors with relative importance higher than 50% (i.e. the relative cumulative Akaike weight for the set of models with ΔAIC < 4) are considered as significant contributors to GD_p_. Color code indicates “non-significant” predictors (RI < 50%, white bars), “significant” predictors not depending on phylum or on the relative position of the population (RI > 50%, black bars) and “significant” predictors depending either on phylum or on the relative position of the population (RI > 50%, grey bars). When bars are absent the relative importance of the predictor is 0%. Please see Tables S8 and S9 for all models with ΔAIC < 4. Source data are provided as a Source Data file.

**Fig. 4:**
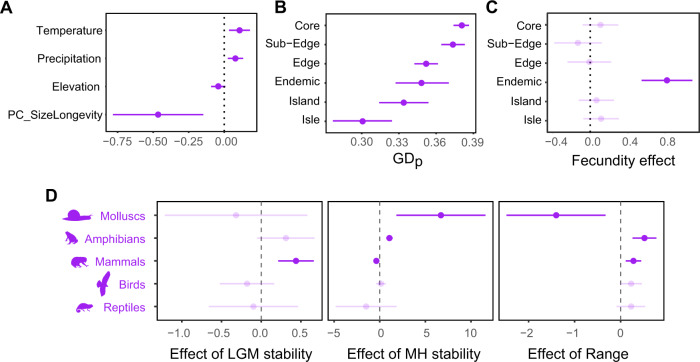
Effects of fixed variables on animal GD_P_. The panels represent 95% confidence intervals with mean phylum-independent effects of temperature, precipitation, elevation and body size/longevity (**A**), biogeographic position (**B**), position-dependent effects of fecundity (**C**) and phylum-dependent effects of temperature stability and species’ range (**D**), as derived from the animal kingdom model (Supplementary Data 6). Non-significant effects (p-values > 0.05) are transparent. Source data are provided as a Source Data file.

For animals, some predictors (Precipitation, Temperature, Elevation and the principal component synthetizing body size and longevity -PC_SizeLongevity-) had a systematic and significant impact on GD_P_ across all phyla, whereas other effects were phylum-specific (MH and LGM temperatures stability, species range) (Fig. 3A). In particular, GD_P_ was higher for populations living in areas with high levels of precipitation and with high temperature, and tended to be higher at low elevation (Fig. 4A). In addition, GD_P_ was higher for small and short-lived species (r-like strategy) (Fig. 4A). The relative position of an animal population within the species range also had a marked influence on GD_P_ (Fig. 4B, Supplementary Data 6). Consistent with the core-periphery hypothesis, animal GD_P_ was highest in core populations and gradually decreased towards the edge of the species range (Fig. 4B). Endemic animal species had surprisingly high GD_P_ levels, similar to GD_P_ levels of edge populations in more widespread species. Species with high fecundity finally had significantly higher GD_P_ than species with low fecundity, but only in endemic species (Fig. 4C, significant interaction term between PC_Fecundity and the position of populations within their range, Supplementary Data 6). As opposed to these general, phylum-independent effects, effects of temperature stability and species range on GD_P_ were heterogeneous across phyla (Fig. 4D). Specifically, there was a significant increase in GD_P_ with increasing long-term temperature stability in amphibians and molluscs since the Mid Holocene (Fig. 4D), and in mammals since the LGM (Fig. 4D). In addition, GD_P_ tended to be higher in species with large distribution ranges, particularly in amphibians and mammals, whereas this relationship was not significant in other phyla, except for molluscs in which it was negative (Fig. 4D).

**Fig. 5:**
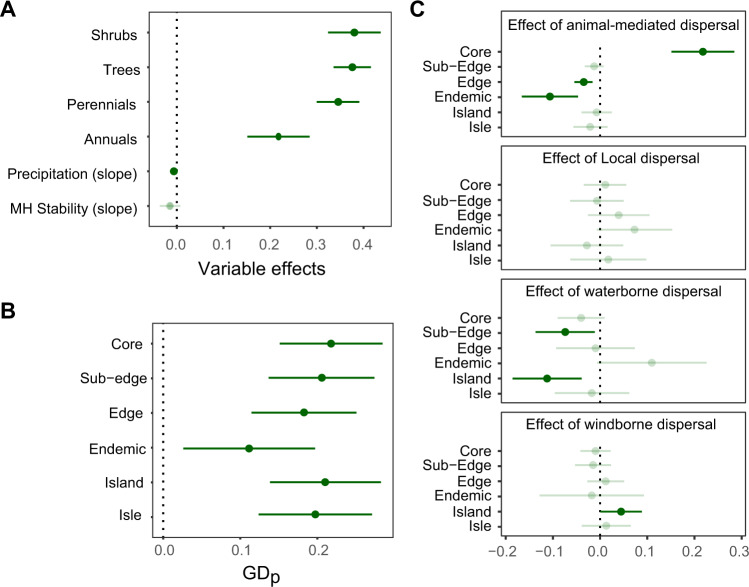
Effects of variables on plant GD_P_. The panels represent 95% confidence intervals with mean phylum-independent effects of lifeform, precipitation and climate stability (**A**), biogeographic position (**B**) and seed dispersal-dependent effects of biogeographic position (**C**), as derived from the plant kingdom model (Supplementary Data 7). Non-significant effects (p-values > 0.05) are transparent. Source data are provided as a Source Data file.

In plants, patterns were different from those in animals. First, all variables affecting plant GD_P_ were consistent across phyla (no interactions of variables with phylum), including precipitation and lifeform that were among the most important variables (Fig. 3B). In particular, highest GD_P_ values were found in the driest climates (Fig. 5A), and GD_P_ increased with lifespan from short-living annuals to long-living trees (Fig. 5A). Temperature stability since the Mid Holocene marginally and negatively influenced plant GD_p_, but this effect was less important than current climate (precipitation) (Fig. 5A, Supplementary Data 7).

As opposed to animal GD_P_, plant GD_P_ was lowest for endemic species and highest in core populations, with a subtle decrease in GD_P_ towards the edges of species’ ranges (Fig. 5B). Insular populations (isles, islands) had similar GD_p_ as those sampled in the core of the range (Fig. 5B). Nonetheless, GD_P_ variation was also explained by a marked interaction between seed dispersal and biogeographic position, indicating that the broad pattern (GD_p_ higher in core populations than in edge populations and endemic species) was mainly driven by plant species with a seed dispersal sustained by animal movements (Fig. 5C). Moreover, this interaction term indicates that island populations have markedly low vs. high GD_P_ for water- and wind-mediated dispersal, respectively (Fig. 5C).

## Discussion

Our study reveals worldwide patterns and underlying drivers of genetic diversity at the local population scale across a broad range of animal and plants species. We first discuss the broad patterns of plant and animal population genetic diversity, as well as the taxonomic generality of the core-edge hypothesis. Specifically, we found that core populations generally harbour higher genetic diversity than edge populations. Nonetheless, in some species groups, core and edge populations sustain similar levels of genetic diversity, which has important implications for conservation management. We further observed phylum-specific impacts of temperature stability, life-history traits, and biogeographical position, which will be discussed for plants and animals separately. We finally examine conservation implications, in addition to limits and prospects for future studies.

A notable pattern revealed by our study is the difference in population genetic diversity between plants and animals, with plants sustaining consistently lower population genetic diversity than animals (Fig. 2A). Several findings arising from our study support a role for different mating systems in explaining this plant-animal discrepancy in population genetic diversity. First, all vertebrates in our study mate through outcrossing, which positively affects effective population size and thus population genetic diversity^18,56,57^. Second, although the breeding system of most mollusc species is poorly known, this phylum is known to contain many self-fertilizing species^58^. Correspondingly, molluscs had low population genetic diversity compared to most other animal phyla (Fig. 2A). Furthermore, where animals used in this study can actively move in search of a compatible mate, the passive nature of pollen and seed dispersal may further reduce plant population genetic diversity.

Our results provide surprisingly weak support for the frequently hypothesized relationship between the distance to equator (expressed as absolute latitude) and genetic diversity^30,44,45,48,51^. Overall, there was no latitudinal gradient of population genetic diversity in plants, and a weak gradient in animals. Similarly, and contrary to the expectation that stable climates at low absolute latitude result in high population genetic diversity due to long-term population persistence, we found little evidence that past temperature stability favours population genetic diversity (Figs. 4A and 5A, but see the next paragraph for phylum-specific patterns). This may suggest that (i) contemporary processes are more important than postglacial recolonization dynamics in explaining population genetic diversity, and/or (ii) microrefugia that are uncoupled from general macroclimatic clines contribute more than macrorefugia to past population dynamics^59^. Importantly, our results are in line with a recent study that could not find clear latitudinal patterns in population genetic diversity for 600 vertebrate species^60^. The strong discrepancy between studies assessing latitudinal gradients in genetic diversity (ref. ^60^ and our study vs refs. ^.^^44,48,51^) calls for a new paradigm regarding the worldwide distribution of population genetic diversity.

Our results support to some extent the core-periphery hypothesis, a major biogeographic theory predicting higher genetic diversity in the core than in the periphery of species’ ranges. Importantly, the decline in population genetic diversity from core to edge habitats seems moderated by dispersal (in plants) and fecundity (in animals). In addition, insular populations did not systematically harbour low population genetic diversity compared to mainland populations, except in animal species (Fig. 4B). Thus, animal species seem to be more restricted by insularity than plant species. Our finding that core populations frequently, but not systematically display higher genetic diversity than edge populations is of major importance as (i) it demonstrates that edge populations can represent non-negligible sources of intraspecific diversity (mostly in plant species, Fig. 5C), and (ii) it emphasizes the discrepancy between GD_P_ and GD_S_, with GD_P_ being sensitive to the biogeographic context of the population. Conservation-wise, edge populations should be considered in light of the species’ life-history traits, or genetically compared to core populations to avoid potential loss of unique genetic variation by prioritizing core populations in conservation planning.

Noteworthy, in both plant and animals, a non-negligible amount of variance in population genetic diversity was explained by the random “species” effect (ca. 70% and 40%, respectively, Tables S6 and S7), representing non-independence due to relatedness as well as study-specific methodological choices and sampling scale. Results of individual studies should thus always be interpreted in light of their methodological approach^61,62^. Here, accounting for molecular marker types and species as weighing and random factors in the models allowed revealing patterns of GD_P_ variation independent of this methodological noise. Nonetheless, it is also possible that the “species” random term aggregates unmeasured characteristics of species that might be important for explaining population genetic diversity (and species genetic diversity), and we call for future studies investigating further these potential unmeasured variables. In parallel, the high species specificity of GD_P_ emphasizes the importance of accumulating more data on individual species for further understanding which taxonomic GD_P_ patterns are key to conservation planning.

Animal population genetic diversity varied substantially among phyla, with reptiles, mammals and birds sustaining particularly high levels of genetic diversity. While we demonstrated that population genetic diversity decreases with latitude for amphibians and molluscs, no trends were observed for other animal phyla (Fig. 2B, Supplementary Data 5). The negative relationship between population genetic diversity and latitude in amphibians and molluscs conforms with the positive relationship between their population genetic diversity and temperature stability since the Mid-Holocene (Fig. 4D), which provides support for the hypothesis that latitudinal gradients of population genetic diversity likely result from longer term population persistence associated with more stable climates in the past^42,63^. Through hampering postglacial movement, limited dispersal abilities (range effect in Fig. 4D) in particular may play a critical role in driving effective population size in amphibians and molluscs in response to past climatic conditions^48,64–68^ (Fig. 4D). Limited dispersal may also explain why we did not find impacts of earlier climate stability (since LGM) on GD_P_ in molluscs and amphibians. Specifically, slow movement may prevent these particular groups of organisms from keeping pace with past climate change, causing rapid and range-wide population turnover, thereby erasing earlier signatures of climate stability on GD_P_ (e.g. between LGM and MH). As the only vertebrate group with a positive relationship between LGM temperature stability and population genetic diversity, mammals seem to manifest the longest-lasting imprint of temperature stability on population genetic diversity_._ This result suggests that mammals exerted relatively rapid postglacial recolonization, explaining why mammal population genetic diversity coincides with LGM rather than with MH climate stability.

In addition to historical climate, we observed a positive impact of precipitation and temperature as independent contemporary climate drivers of population genetic diversity, indicating that analogously to species richness, highly productive (tropical) ecosystems can carry larger animal populations and are less prone to genetic drift^69–71^. Independent from these climatic effects, we found a weak, but expected, negative relation between population genetic diversity and elevation, suggesting that populations are more isolated at higher elevation. This finding is in line with the core-periphery effect that was most pronounced in the animal kingdom (Fig. 4B).

Endemic animal species appear to harbour a surprisingly high amount of genetic diversity within their populations despite their limited geographic range. The genetic signature of natural rarity has commonly been investigated in plant species, where it has been shown that endemic species frequently, but not systematically, harbour relatively high levels of population genetic diversity and resistance to habitat fragmentation^57,72,73^. In compliance with these studies, we argue that species that are naturally rare with respect to their geographic range can still reach high-effective population sizes, possibly because their populations have been historically more stable and less affected by postglacial colonization dynamics. This seems to be especially valid for highly fecund, endemic animal species, which exhibited particularly high population genetic diversity (Fig. 4C).

Large and long-living animal species harbored significantly lower population genetic diversity than small, short-living species (Fig. 4A). This is in line with earlier studies, which attributed this effect to higher rates of molecular evolution in small-bodied animals as compared to large animals^22,24^. Likely, large animal species also have experienced increased anthropogenic pressures, for example through hunting and fragmentation of their large population territories^74^.

Among the major plant groups, only the eudicots were found to show a significant (yet weak) correlation between population genetic diversity and distance to the equator (absolute latitude in Fig. 2B). However, contrary to expectations, population genetic diversity increased with distance to the equator in this phylum. We suspect that (i) lower interspecific competition away from the equator^75–77^ may allow more successful population establishment of species adapted to more stressful conditions, and/or (ii) stressful environments away from the equator trigger a shift in energy allocation from individual growth to reproductive output, thereby increasing population genetic diversity. While stressful environments may favour reproductive output (e.g. fruit production), a meta-analysis based on 164 published studies rejected the hypothesis that drought stress triggers a shift in energy allocation from biomass to reproductive tissues^78^. Interspecific competition thus represents a more likely driver of global patterns of population genetic diversity. We also identified a consistent and significant—but weak—negative relationship between precipitation and plant population genetic diversity, reinforcing the notion that stressful (dry) environments might favour high levels of genetic diversity in plant populations (Fig. 5A). While this relationship is the reverse of the positive relationship between precipitation and animal population genetic diversity, the contribution of precipitation to both plant and animal population genetic diversity emphasizes the role of precipitation as a major evolutionary force. This finding adds to the evolutionary significance of precipitation as the dominant driver of natural selection in animal and plant populations for over 150 species^79^.

Plant species with wind-dispersed seeds achieved particularly high levels of population genetic diversity on islands (Fig. 5C), demonstrating that, in contrast to the expectation that geographic isolation and limited habitat availability decrease population genetic diversity, specific life-history traits may actually boost population genetic diversity on islands to levels that exceed mainland population genetic diversity. Wind-directed dispersal may thus favour successful establishment and persistence in isolated environments, such as islands and isles^80,81^. This finding suggests that populations of plant species adopting seed features adapted to wind dispersal are less prone to genetic drift than species relying on other dispersal strategies. Unexpectedly, water dispersal seems to be a particularly unsuccessful mechanism in insular conditions (Fig. 5C), where downstream river dispersal back to the ocean constrains seedling establishment and subsequent population growth.

Interestingly, while a general but subtle decrease in population genetic diversity was observed towards the edge of plant distribution ranges, the core-periphery effect was markedly pronounced for plant species featured by animal-dispersed seeds (Fig. 5C). This result likely arises from the scattered distribution of suitable habitat for plants towards the edge of their distribution, which may particularly hamper the establishment and maintenance of plant populations where animals are required to transport seeds between isolated patches of suitable habitat. The important role of animal-mediated dispersal in mitigating the spatial distribution of plant population genetic diversity suggests that habitat fragmentation and configuration may particularly affect the group of plant species that depends upon animals for exchanging genetic material.

Our finding that plant lifeform is associated with population genetic diversity, with long-living species showing highest levels of GD_P_, likely reflects the expected impact of habitat fragmentation and reconfiguration on global patterns of GD_P_, since long-living plants respond more slowly to reduced gene flow. This extinction debt, predominantly linked to woody species, has been of considerable concern to nature conservation, and indicates an underestimation of the number of long-living species endangered by habitat fragmentation^21,82–84^. Although we did not specifically collect studies in the context of habitat fragmentation, land-use change and consequent fragmentation are among the most pronounced global change drivers across the earth, and have most likely affected most populations to some extent^85,86^.

Our global synthesis shows that populations can harbour naturally low levels of genetic diversity (e.g. plant as compared to animal populations), seemingly driven by species’ life-history traits, by the location of a population relative to the species’ distribution range, and by contemporary climate. A given level of population genetic diversity will thus have different conservation implications for different populations even if they belong to the same species, and low population genetic diversity may not necessarily translate into low fitness or low evolutionary potential^87,88^. As a result, any attempt to assess the effects of environmental stressors on population genetic diversity should consider the expected baseline levels of population genetic diversity for similar populations not exposed to these stressors.

It has been demonstrated that regional genetic diversity at the species level (Supplementary Notes 1) decreases toward the poles in amphibians, mammals and fish^44,48,51^; an effect attributed to temperature-dependent mutation and diversification rates. Our results illustrate that population genetic diversity (GD_P_) and genetic diversity at the species level (GD_S_) can have strongly different spatial patterns, likely as a result of the interplay between biogeography, climate and species traits together shaping local effective population size. This result has strong implications for the management of local populations with distinct evolutionary histories (cfr. ESUs, see also Supplementary Notes 1). We demonstrate that the local biogeographic properties of a population are much more important determinants of effective population size than range size, a commonly used indicator of population size (IUCN). For example, populations of endemic species achieve levels of genetic diversity that can exceed population genetic diversity in more widespread species (Fig. 4B and C), indicating that effective population sizes are in many circumstances uncoupled from species’ range sizes.

Our findings demonstrate great potential for generating a unified conservation genomics framework for biodiversity monitoring and prioritization that considers population genetic diversity across space and species. Such a universal perspective on spatial and cross-taxon population genetic diversity patterns becomes particularly appealing with the increasing use of SNPs to calculate population genetic diversity, and opens the door for exploring population genetic diversity -extinction risk associations across taxa to support genetic marker-based conservation assessment.

We used expected heterozygosity of neutral nuclear markers, which is the most widely used index of genetic diversity, to explain population genetic diversity patterns across space and taxa. A major improvement would be a similar exercise that uses (i) allelic richness, (ii) adaptive genetic variation, or (iii) epigenetic variation as response variables. First, allelic richness has been suggested to respond more readily to anthropogenic stressors, and may therefore be particularly useful when addressing questions related to human-mediated changes in genetic diversity^7,86^. Second, adaptive genetic variation as identified through landscape genomic analyses (i.e. environment association studies)^89,90^ is related to the adaptive potential of natural populations. Thus, while neutral genetic diversity is informative with respect to inbreeding and demographic processes, adaptive genetic diversity is indicative of long-term adaptive potential and persistence in light of environmental changes. Third, epigenetic variation has been increasingly shown to be tightly linked to environmental variation^91–93^. How epigenetic variation is driven by biogeography, life-history, climate and anthropogenic stressors, however, remains unexplored and its assessment could reveal novel insights into the spatial distribution, determinants and conservation implications of molecular diversity.

The amount of variance in GD_P_ explained by molecular marker and study effects (captured by our “species” variable) demonstrate that a more standardized methodology is crucial for allowing comparison between species and studies. The enormous discrepancy in population genetic diversity estimates among studies questions their use in biodiversity conservation, and compromises the development and operationalization of a unified population genetic diversity framework for monitoring global biodiversity^94,95^. The ongoing transformation from population genetics into population genomics is nevertheless promising, since individual outlier SNPs typically have a much more reduced impact on population genetic diversity estimates than microsatellite loci with suspicious allele distributions. To further ensure the comparability and usefulness of published population genetic diversity metrics, a detailed description of the study species and of the geographical position of the sampled populations helps contextualizing and comparing estimates of genetic diversity.

To further elucidate the global drivers of GD_P_, additional traits may deserve more attention in future projects, including animal gamete dispersal (e.g. pelagic duration of larvae in molluscs and other animal taxa^96–98^), and strategies of space use (incl. home range dynamics, territoriality, nomadism, dispersal and migratory behaviour^99–101^). For example, particularly low dispersal abilities and high habitat specificity within the amphibian clade may partly explain the generally low genetic diversity in amphibian species (Fig. 2B)^66^. These traits are, however, unknown for a large number of species, preventing us from including them in our models. In plants, seed bank persistence^102,103^ and fecundity-related traits (e.g. average seed production) may be strong moderators of population genetic diversity. Indeed, the important role of fecundity in the animal kingdom suggests that the low amount of explained variance of plant GD_P_ may be due to the lack of fecundity metrics in our plant models. Seed set is correspondingly considered an important fitness trait^104^, with inbreeding frequently resulting in reduced seed set^105^. The exploration of anthropogenic impacts on population genetic diversity may finally uncover additional global patterns, and allows accounting for anthropogenic effects while establishing natural baseline population genetic diversity values.

Comprehensively considering the wide array of factors that can simultaneously and interactively affect genetic diversity at the local (population) scale is crucial for understanding the processes affecting evolutionary dynamics and long-term sustainability of populations. Here, we provide a global map of within-population genetic diversity, and we demonstrate surprisingly distinct patterns and drivers of population genetic diversity between plants and animals. We show that patterns of genetic diversity at the population scale (linked to effective population size) do not align with those typically identified for genetic diversity measured at the species level (linked to diversification rates), and that genetic diversity at the population scale is shaped by a complex interplay between historical climate, current climate, biogeography and life history. This interplay questions several conventional assumptions, such as the expectation that island, edge and endemic populations systematically harbour low levels of genetic diversity. Our findings have major conservation implications, and raise questions regarding the efficacy of formulating management solely based on genetic diversity measured at the species level or on range-wide measures of population size. Instead, our results encourage the development of a conservation framework based on genetic diversity measured at the population scale and accounting for biogeographic context and life-history traits.

## Methods

### Response variable

Expected heterozygosity (hereafter GD_P_)^106^ was chosen as the response variable as it is the most studied index of within-population genetic diversity, fundamentally descriptive and consequently predicted to be relatively insensitive to publication biases. Moreover, it is directly related to effective population size^107,108^. Although expected heterozygosity may be slower at responding to recent demographic setbacks than allelic richness, it is less sensitive to difference in sample size. Moreover, recent longitudinal studies have shown that H_E_ is sensitive to anthropogenic disturbance, and that significant reductions in expected heterozygosity can be observed several generations after a population bottleneck^109,110^. For example, a South-African lion population suffered a 10–13% reduction in expected heterozygosity as compared to museum samples collected 100 years earlier (ca. 14 generations), even though the census population remained locally abundant^110^. This demonstrates the importance of considering expected heterozygosity as a proxy of GD_P_ in addition to census population counts for a more accurate assessment of a population’s conservation status.

Because GD_P_ is very sensitive to marker type (e.g. GD_P_ is restricted between 0 and 0.5 in AFLP markers and between 0 and 1 in microsatellite markers), GD_P_ values from each marker type were standardized (mean = 0 and variance = 1) to make them comparable across studies. Standardized GD_P_ values were then normalized as (GD_P__scaled-min)/(max-min) to range from 0 to 1.

### Data collection

Using the terms “expected heterozygosity” AND “genetic marker” AND “populations” AND “plant” OR “amphibian” OR “reptile” OR “bird” OR “mammal” OR “mollusc”, from 2000 up to 2015, we searched for articles that estimated population genetic diversity using Google Scholar. Taxonomic variants (e.g. “cephalopod” in addition to “mollusc”) are specified in the PRISMA diagram provided in Supplementary Fig. 1. We only included studies that were representative for natural genetic diversity, i.e. we eliminated articles involving introduced species, invasive populations and cultivars. We primarily focused on multi-population studies in order to capture within-species biogeographic variation. Studies that did not provide a map or coordinates for each population were excluded, and so were populations with sample sizes lower than 10 individuals. We focused on terrestrial and freshwater processes and therefore excluded all marine populations.

### Predictors of GD_P_ variation

For each population, we collected information on the geography, climatic conditions (past and contemporary), biogeography and species life history. The geographical coordinates of each population with a GD_P_ estimate were extracted using the general WGS84 coordinate system to obtain the spatial variables “Longitude”, “Latitude” and “|Latitude|”, the latter reflecting the distance from the equator. We downloaded georeferenced raster files to characterize local and current climatic data (worldclim.org) in the form of the three synthetic predictors “Temperature”, “Precipitation” and “Humidity” using a principal component analysis (see Supplementary Methods). In addition, because historical climate variability may have imprinted GD_P_ (populations may have persisted much longer in regions featured by stable climates as opposed to more variable climates where population turnover and bottlenecks are more frequent), we used averaged temperatures for the Mid-Holocene climate (MH; the last 6000 years from now) and for the Last Glacial Maximum climate (LGM, the last 22,000 years from now), and calculated “MH stability” and “LGM stability” as the standardized differences between the current temperature and the past temperature calculated either from LGM or MH, respectively, (see Supplementary Methods). We classified the biogeographic position of each population (“Position”) on the mainland as edge (the outer 25% of the species’ distribution area), subedge (25–50%), core (within 50% of the species’ distribution area) or endemic (species restricted to an area <10.000 km²). “Position” also included the classes island (>10.000 km²) and isle (<10.000 km²) to account for effects of isolation and area restrictions on GD_P_. “Elevation” was also retrieved for each population. Although elevation encompasses climatic variation among populations, climatic variation is accounted for by the three climatic variables described above. Remaining elevation effects are therefore considered as a proxy for the isolation of populations. We expected GD_P_ to be lower at higher elevation due to an increase in spatial isolation (i.e. decrease in gene flow).

For each species, we obtained information on life-history traits and distribution ranges from iucn.org, utheria.org, animaldiversity.org, try-db.org, eol.org and the source papers used for collecting H_E_ (Supplementary Data 3). To characterize animal life history, we focused on three life-history covariates (log10-transformed): life-time “Fecundity”, average body “Size” and maximum “Longevity” (Supplementary Fig. 2, Supplementary Methods). To reduce collinearity among these life-history traits, data were synthetized into two principal components (R package *vegan*), the first one (hereafter “PC_SizeLongevity”) being positively associated with Size and Longevity and the second one (hereafter “PC_Fecundity”) being positively associated with Fecundity (Supplementary Methods). To characterize plant life history, we included gamete dispersal (“Pollen”: biotic [*n* = 2341 populations] vs. abiotic [1913]), zygote dispersal (“Seed”: animal [1398] vs. wind [1779] vs. water [497] vs. *local* [580]), “Mating” (self-incompatible [1731] vs. self-compatible [1408] vs. clonal [753] vs. non-clonal [a group comprising the 363 remaining populations]), and “Lifeform” (*annuals* [164] vs. perennials [1405] vs. shrubs [308] vs. trees [2337]) (Supplementary Methods). The number of classes in categorical variables was limited to avoid model overfitting. As a proxy for a species’ habitat specificity and/or dispersal capacity, we used “Range”, representing the size of a species’ distribution. A detailed account of the composition of all environmental, life history and geographical variables, as well as the Pearson correlation coefficients between all covariates (all < 0.6), are provided in Supplementary Methods and Supplementary Tables S1–S3.

In all models, we (i) weighted model residuals by the number of markers and sample sizes through a frequently used weighing factor in meta-regressions^111^ and allowing to take into account the precision of the estimate (more weight is given to estimates with a higher precision, i.e. with higher sample size and number of loci)

$$1/\sqrt {{\mathrm{log}}\left( {{\mathrm{Loci}} \,\times\, {\mathrm{SampleSize}}} \right)},$$

(ii) included “Marker” (co-dominant markers vs. dominant markers vs. enzymes) to control for non-independence within marker types, and (iii) incorporated “Species” (extracted using the R package Taxize) to control for non-independence due to both phylogenetic relatedness and study-specific methodological aspects such as H_E_ estimation methods and sampling protocols.

### Modelling

In a first, descriptive step, we aimed to test whether plant and animal species have distinct levels of GD_P_ across the globe. Because distance to equator is thought to be an important moderator of genetic diversity through its association with temperature, productivity, long-term stability and historical range expansions, we implemented a linear mixed modelling (LMM) approach testing the impacts of |Latitude|*Kingdom on GD_P_ while controlling for non-independence within marker types and species (see above).

We then assessed whether GD_P_ varied across broad taxonomic groups within kingdoms, i.e. for plants (eudicots vs. monocots vs. magnolids vs. pines) and animals (mammals vs. birds vs. reptiles vs. amphibians vs. molluscs) separately. To this end, we replaced the “Kingdom” predictor by a “Phylum” predictor in the LMM described above, and added the “Kingdom” effect as a random term to the model to account for non-independence within the plant and animal kingdoms.

In a second, more comprehensive step, the mediating role of life-history traits, current climate, past climate stability and biogeography on GD_P_ was modelled, first for animals (animal kingdom model) and then for plants (plant kingdom model). Because the impact of life history on GD_P_ may depend on the biogeographic position of a population, we included the pairwise interactions between position and each life-history variable. In addition, we assessed whether the effects of life-history traits, current climate, climate stability, elevation and biogeography on GD_P_ were consistent across phyla, and thus included all possible pairwise interactions with “Phylum”. Species with unknown life-history traits were excluded from the models, leaving 2544 data points (229 species) for the animal kingdom model and 4254 (308 species) for the plant kingdom model. Because life-history trait information is extremely scarce for molluscs, this phylum was poorly represented in the animal kingdom model (Supplementary Fig. 2). Model assumptions (normality and heteroscedasticity of residuals) and model fits (residual vs. fit plots) and publication biases were evaluated visually. Residual and funnel plots did not point to considerable biases (Supplementary Tables S4–S7, Supplementary Methods, Supplementary Fig. 3). Modelling and model evaluation were performed in the R environment (R package *glmmTMB*).

All possible variants of the full animal and plant kingdom models were tested using the ‘dredge’ function in the R package *MuMln*, and the relative importance (RI) of all simple and interaction terms was quantified as the sum of the AIC weights across all the models with a ΔAICc < 4 (26 models for animals and 116 models for plants, see Tables S8 and S9) in the set where each term occurs^112^. We standardized this sum by the total AIC weight of all models with a ΔAICc < 4 so that the RI of each term varied between 0 and 100%. When the RI of a term was 100% it means that the term was included in all models with a ΔAICc < 4. We arbitrarily considered that a term was biologically relevant (and was hence interpreted) when RI > 50%, and these terms were included in the final models used to infer estimates associated to each term. The proportion of variance explained by the final model’s fixed effects (R²_m_, or marginal R²) and the proportion of variance explained by both the fixed and random factors (R²_c_, or conditional R²) were obtained using the R package *MuMln*.

To test for spatial autocorrelation, we used an autocorrelogram approach assessing the relationship between Moran’s I of model residuals and pairwise geographical distance^109^. We did not find evidence for patterns of spatial autocorrelation neither for the animal kingdom model nor for the plant kingdom model (Supplementary Fig. 4, Moran’I was weak whatever the class of distance), indicating that spatial autocorrelation is unlikely to impact estimate inferences of these models^110^. We therefore did not include spatial terms in the animal and plant models^110^. Interestingly, we performed the same procedure on the raw GD_P_ data (Supplementary Fig. 4) and we only identified spatial autocorrelation at a very fine spatial scale for animals and to a lesser extent for plants.

To test the extent to which the spatial (geography) and random (species) structure of our model may affect model parameter robustness due to potential overfitting, we consecutively left out all data points from (i) each random species (219 in animals and 306 in plants) or (ii) each occupied squared geographical area (3 degrees latitude and longitude; 57 areas in animals and 75 areas in plants) using a jacknife procedure, to obtain 95% confidence intervals about each parameter. Our models appear highly robust to both geographic and taxonomic variations (Supplementary Fig. 5).

### Reporting summary

Further information on research design is available in the Nature Research Reporting Summary linked to this article.

## Supplementary information

Supplementary Information

Peer Review File

Reporting Summary

Description of Additional Supplementary Files

Supplementary Data 1–9

## Data Availability

All datasets supporting the results are deposited on Figshare (10.6084/m9.figshare.13373363). Source data are provided with this paper.

## Source data

Source Data

## Code availability

The R scripts supporting the results are available on Figshare (10.6084/m9.figshare.13373363).
