# Supplementary material for: Life history, climate and biogeography interactively affect worldwide genetic diversity of plant and animal populations
Source: Nat Commun. 2021 Jan 22;12:516. doi: 10.1038/s41467-021-20958-2 (PMC7822833; doi:10.1038/s41467-021-20958-2)
Supplement: Supplementary file 1 — Supplementary Information [file 41467_2021_20958_MOESM1_ESM.pdf]

Life history, climate and biogeography interactively affect worldwide genetic diversity of plant and animal populations

H. De Kort**, J.G. Prunier, S. Ducatez, O. Honnay, M. Baguette, V.M. Stevens, S. Blanchet

*** Correspondence to:** hanne.dekort@kuleuven.be

Supplementary Notes (pp 2)

Supplementary Methods (pp 3-4)

Supplementary Tables (pp 5)

Supplementary Figures (pp 6-12**)**

Supplementary Notes 1

Population genetic diversity (GD_P) vs. intraspecific genetic diversity (GD_S)

All differences between GD_P (local scale) and GD_S (range-wide scale) can be reduced to the sampling scale, i.e. the scale at which individuals were sampled to calculate GD. Specifically, GD_S does not capture the effects of the local environmental and biogeographic context on individual populations because the sampled individuals originate from distinct populations that evolved independently. GD_P, on the other hand, is calculated for a population of individuals sharing the same eco-evolutionary history. Therefore, GD_P is commonly used in population genetic studies as it captures the effects of the local environment and more contemporary processes (mutation, gene flow, drift and selection), as well as genetic signatures of events in the more distant past. Distinct local populations, each featured by particular allele frequencies, are also referred to as evolutionary significant units (ESUs), i.e. distinct population units that require separate management because they experienced independent evolutionary histories[8].

While similar eco-evolutionary and biological processes may drive both GD_P and GD_S, the outcome of these processes on GD_P and GD_S can be very different. For example, at the periphery of a species distribution, GD_P is expected to be low due to low connectivity to other populations and thus reduced gene flow. The same biogeographic context (i.e. at the distribution edge), however, may increase GD_S across individuals sampled in independent edge populations because these populations have distinct genetic signatures.

Most species are facing population declines at local and regional scales, and can benefit considerably from local conservation efforts that can prevent population extinctions. Therefore, a GD_P baseline (which defines how genetic diversity is predicted to vary according to the environmental and biogeographic properties of populations within and across species) to which conservation practitioners may tailor their conservation strategy, could facilitate local population restoration before extinctions, and eventually, major range contractions occur.

Supplementary Methods

Description of model **variables** and their collinearity

Environmental variables

Elevation and climate data were extracted for the geographical coordinates (WGS84 coordinate system) of each population in the dataset, using QGIS. Please see **Supplementary Fig. 6** for correlation coefficients between each pair of covariates.

- **Elevation**: data extracted from the "The Global Land One-kilometer Base Elevation (GLOBE) Digital Elevation Model, Version 1.0" downloaded from ngdc.noaa.gov.
- **Temperature**: the first principal component (PC) of a principal components analysis (PCA) performed on all bioclim variables downloaded from www.worldclim.org (Hijmans, Cameron, Parra,Jones, & Jarvis, 2005) at ~1 km resolution. This component mainly consisted of bioclim variables related to temperature, and represents 41.11% of the variation across bioclim variables. See Supplementary Table 1 below.
- **Precipitation**: the second PC of the PCA described above (23.57% of variation). See Supplementary Table 1 below.
- **Humidity**: the third PC of the PCA described above (14.12% of variation). See Supplementary Table 1 below.
- **MHC stability**: (|Current average temperature – MHC average temperature|) / (|Current average temperature + MHC average temperature|). Multiplied by (-1). MHC = Mid Holocene Climate.
- **LGM stability**: (|Current average temperature – LGM average temperature|) / (|Current average temperature + LGM average temperature|). Multiplied by (-1). LGM = Last Glacial Maximum.

Life history traits

- **Fecundity:** Life time fecundity log10-transformed and estimated as [maximum life span – age at maturity) * clutch rate (per day) * clutch size (number of offspring)]. We chose maximum instead of average life span because of higher data availability.
- **Size**: Log10-transformed body size (mm)
- **Longevity**: Maximum documented longevity (in days and log10-transformed).
- **PC_SizeLongevity**: The first PC of a PCA performed on the three life history traits Fecundity, Size and Longevity. This component mainly related to Size and Longevity, and represents 49.61% of the variation. See **Supplementary Table 2** below. The principal component is positively correlated to Size and Longevity (see **Supplementary Fig. 7** below).
- **PC_Fecundity:** The second PC of the PCA described above. This component mainly related to Fecundity, and represents 36.85% of the variation. See **Supplementary Table 2** below. The principal component is positively correlated to Fecundity (see biplot below).
- **Range**: obtained from IUCN.org or estimated based on distribution maps (using known sizes of continents and islands as a reference). Range was log10-transformed.
- **Mating**: Plant breeding systems, a discrete variable roughly composed of *self-incompatible* plants (e.g. dioecious plants, herkogamous plants, dichogamous plants), *self-compatible* plants, *clonal* plants (e.g. stoloniferous plants, surculose plants). Plants with unknown mating system, but that are known to be *non-clonal*, were considered as a rest group. Plants with mixed mating systems were considered self-compatible.

- **Pollen**: gamete dispersal, a discrete variable roughly composed of *animal*-dispersed pollen (mostly insect pollination) and *wind*-dispersed pollen (e.g. all grasses).
- **Seed**: zygote dispersal, a discrete variable composed of *animal*-dispersed seed (e.g. seeds with morphological adjustments that allow epizoochorous dispersal, endozoochory, myrmecochory), *wind*-dispersed seeds (e.g. dust (orchid) seed, winged seeds), *local* dispersal (e.g. through ballistics, gravity) and *water*-dispersed seeds (e.g. floatable seeds of shoreline, mangrove and wetland plants).
- **Lifeform**: a discrete variables composed of annual herbs, perennial herbs, shrubs and trees.

Supplementary Tables

Supplementary Table 1. Contribution of each bioclim variable to the three main principal components. PC1 mainly represents **temperature**-related bioclim variables and increases with increasing temperature and temperature stability; PC2 increases with increasing **precipitation**; and PC3 describes the relation between temperature and precipitation, with high values corresponding to high air **humidity** (high temperatures combined with high precipitation).

		**PC1**	**PC2**	**PC3**
BIO4	Temperature Seasonality (standard deviation *100)	-3.7243	0.048123	2.303213
BIO7	Temperature Annual Range (BIO5-BIO6)	-3.4083	-0.83089	2.433579
BIO2	Mean Diurnal Range (Mean of monthly (max temp - min temp))	-0.1591	-2.67092	0.921646
BIO14	Precipitation of Driest Month	0.62814	3.995102	-0.44002
BIO17	Precipitation of Driest Quarter	0.80224	4.015477	-0.45319
BIO15	Precipitation Seasonality (Coefficient of Variation)	1.30027	-2.54642	2.105602
BIO18	Precipitation of Warmest Quarter	1.58941	2.426446	2.977026
BIO19	Precipitation of Coldest Quarter	1.6059	2.644865	-1.30345
BIO8	Mean Temperature of Wettest Quarter	2.51716	-0.89868	2.597652
BIO12	Annual Precipitation	2.66844	3.399593	1.214909
BIO5	Max Temperature of Warmest Month	2.86425	-2.3734	1.490397
BIO16	Precipitation of Wettest Quarter	2.93363	2.202792	2.100313
BIO13	Precipitation of Wettest Month	2.94331	2.027954	2.22948
BIO10	Mean Temperature of Warmest Quarter	3.35716	-1.74248	1.544276
BIO3	Isothermality (BIO2/BIO7) (* 100)	3.47617	-1.1497	-1.13913
BIO9	Mean Temperature of Driest Quarter	3.65747	-1.14981	-1.86088
BIO1	Annual Mean Temperature	4.29228	-1.30854	0.090377
BIO6	Min Temperature of Coldest Month	4.32005	-0.53547	-1.25605
BIO11	Mean Temperature of Coldest Quarter	4.38672	-0.92608	-0.88236

Supplementary Table 2. Contribution of each animal life history variable to the three main principal components. PC1 mainly represents **Longevity and Size** (log-transformed); PC2 increases with increasing **Fecundity**.

	PC1	PC2	PC3
Log_Longevity	-2.2040	-1.5551	-1.117
Log_Fecundity	0.9798	-2.6265	0.816
Log_Size	-2.6209	0.3259	1.245

Supplementary Figures

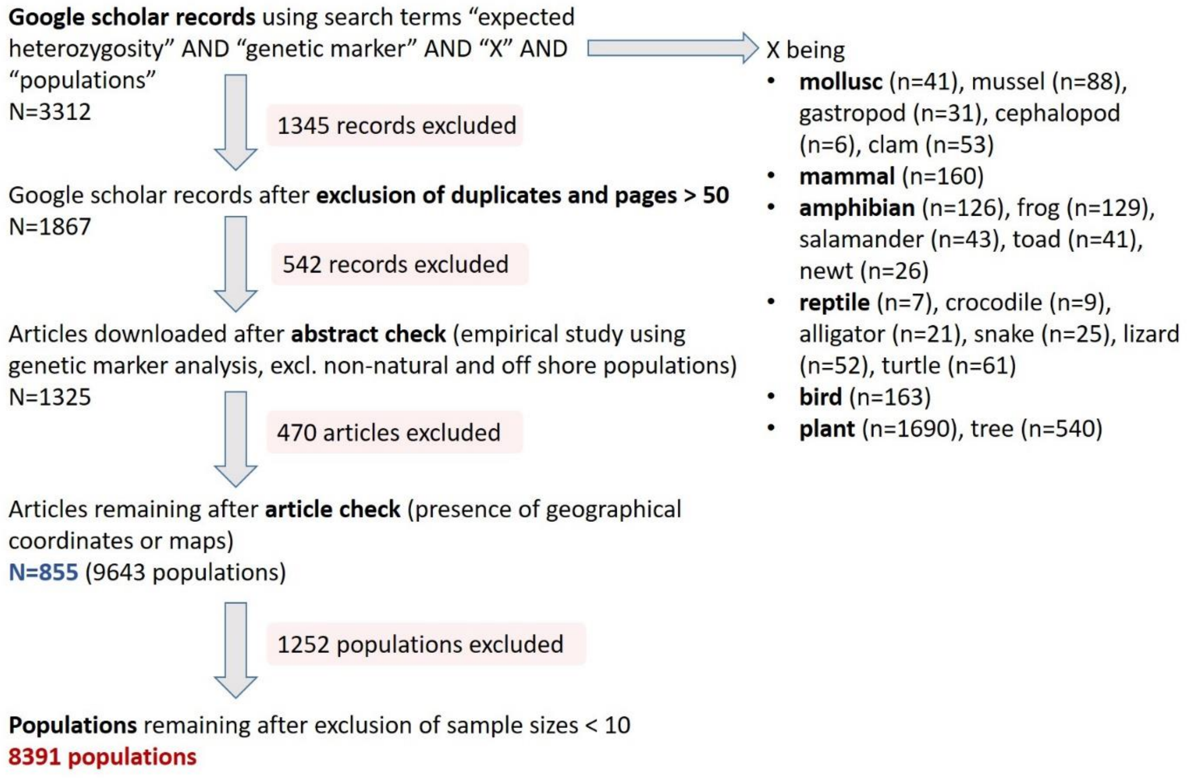

Supplementary Fig. 1. **PRISMA diagram detailing the steps followed during data collection**. Where google scholar records rendered more than 50 pages of records ("plant" "populations" and "animal" "populations"), only the first 50 pages were considered. N and n refer to the total and taxon-specific number of publications.

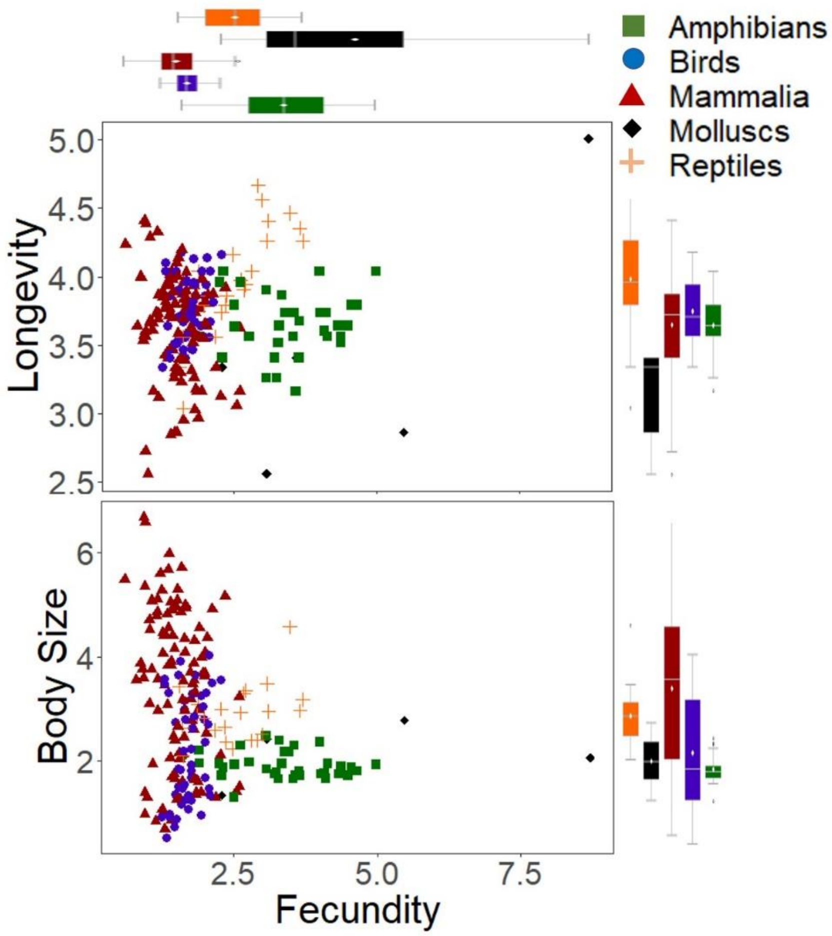

Supplementary Fig. 2. Distribution of life history traits (all log10-transformed) across phyla. The black outlier at the right is the freshwater pearl mussel *Margaritifera margaritifera*, featured by small body size, high fecundity rates and a life span exceeding 200 years. Box plots have same units as corresponding scatter plot axis: (Longevity = log10(days); Body Size = log10(cm); Fecundity = log10(lifetime offspring), and include means (white dot), medians (white dash), 25%-75% quantiles (boxes) and 5%-95% quantiles (whiskers). Sample sizes: N = 33 mollusc, 125 mammal, 71 bird, 34 reptile and 36 amphibian species, resp. See Supplementary Methods for a detailed description of each life history trait. Source data are provided as a Source Data file.

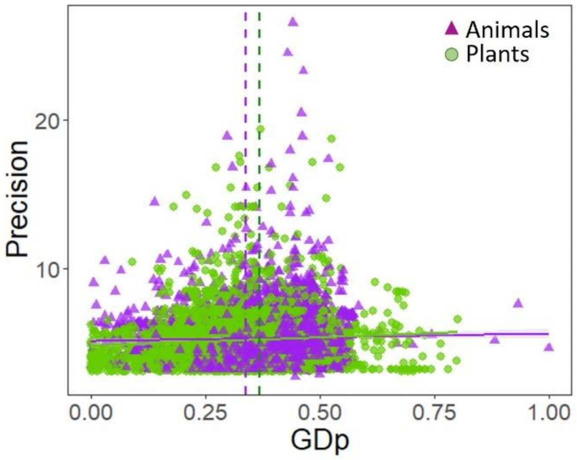

Supplementary Fig. 3. Funnel plot to test for publication bias. The figure shows SQRT Sample Size vs. GD_P_norm for plants (green) and animals (red) and indicates little publication bias. Dashed vertical lines represent averages. Solid regression lines indicate no relation between precision and effect size. Some data points appear skewed towards the upper right corner, but these points represent <1% of all data. Source data are provided as a Source Data file.

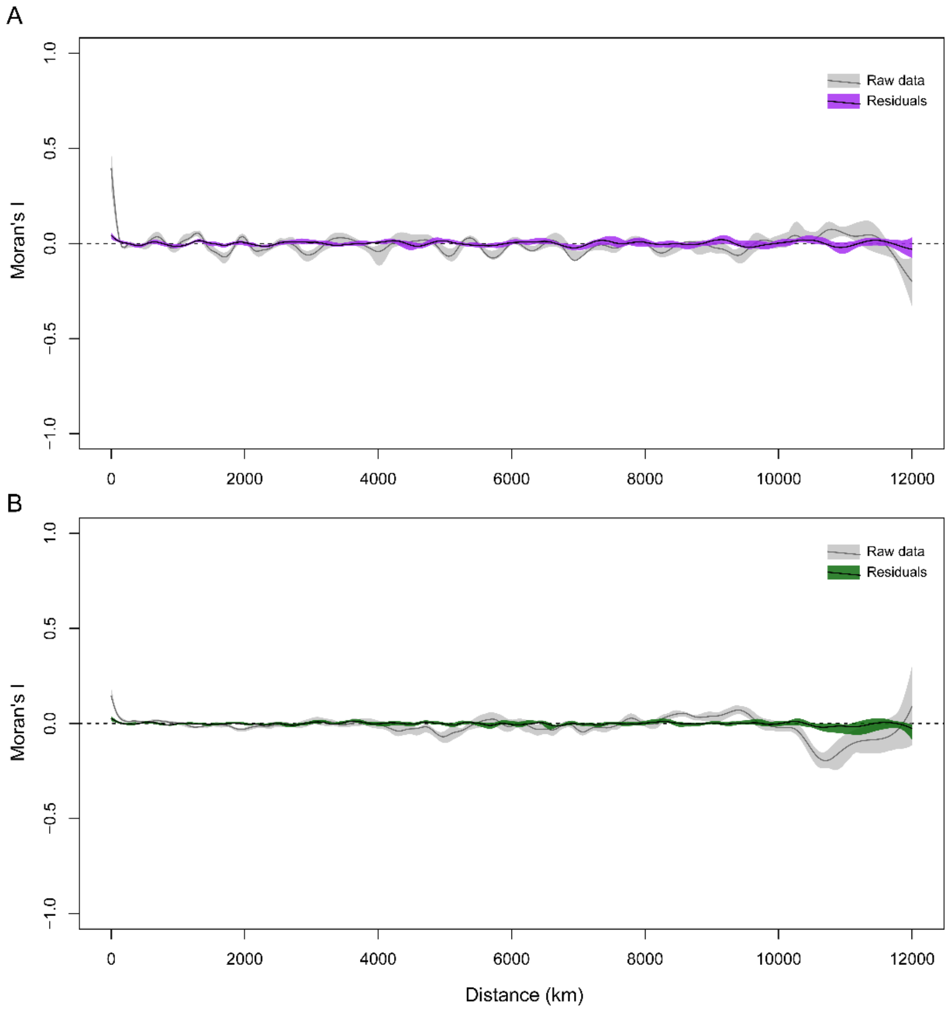

Supplementary Fig. 4. Autocorrelation plots. Panels represent autocorrelograms of Moran's I across pairwise population distances for animals (upper panel) and plants (lower panel) on raw data and model residuals. The near-zero spatial autocorrelation on model residuals across space demonstrates that our model adequately accounts for spatial autocorrelation.

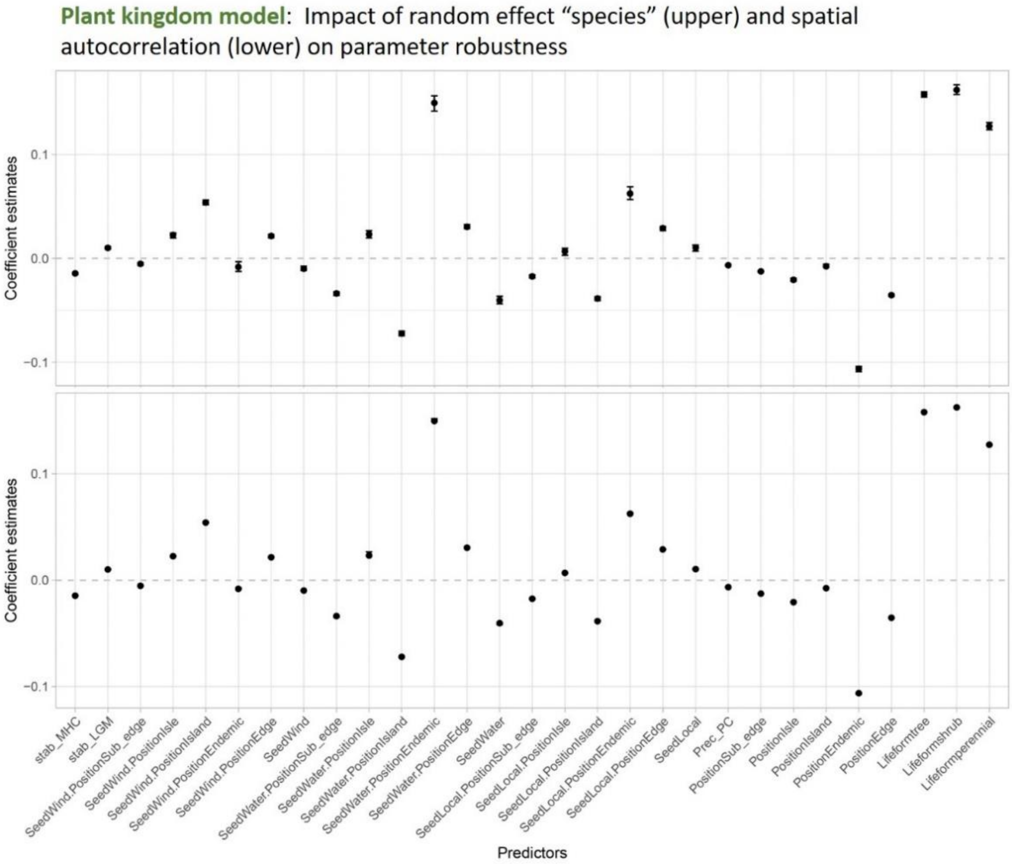

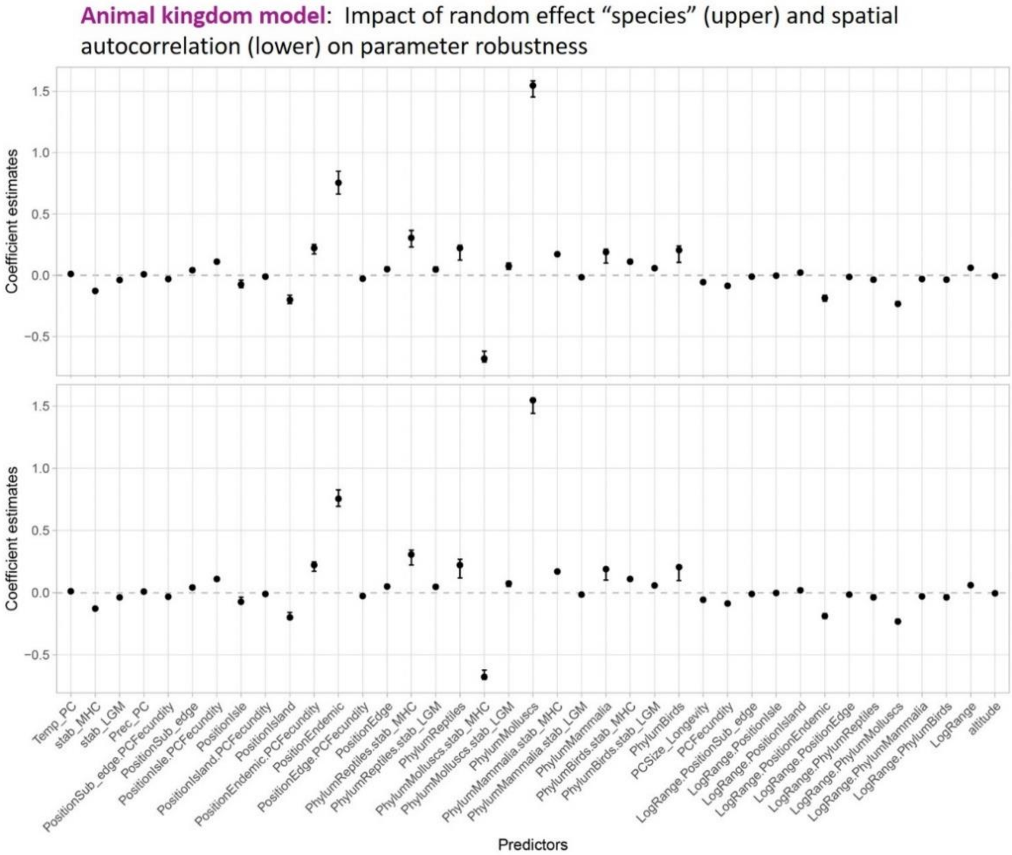

Supplementary Fig. 5. **Jacknife output.** Panels represent model parameter robustness after leaving out random species and geographical areas from plant (upper panels) and animal (lower panels) kingdom model.

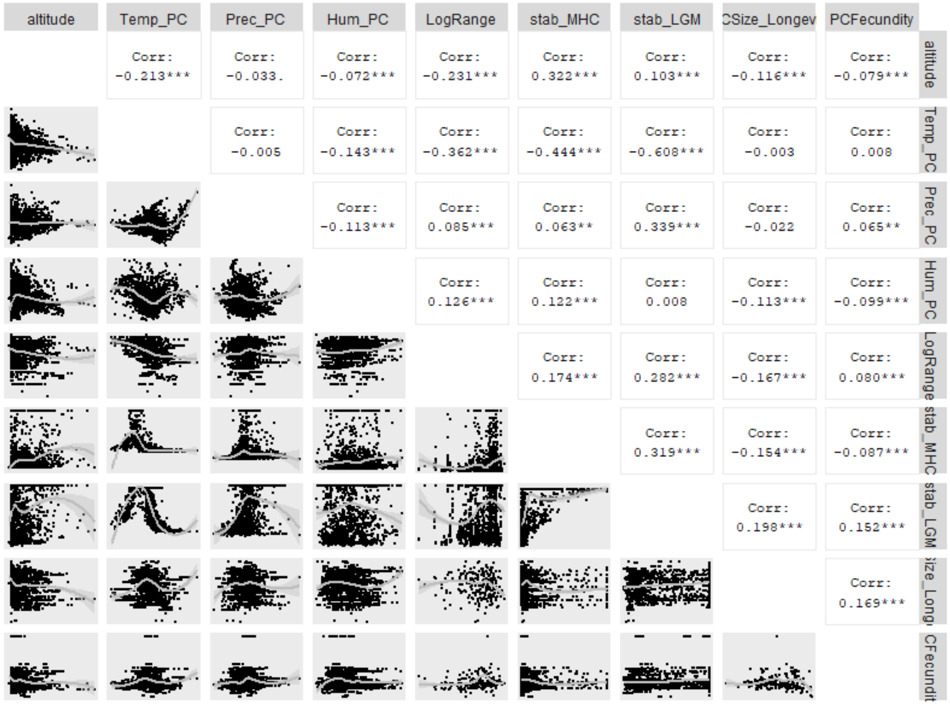

Supplementary Fig. 6. Pairwise correlations between all model covariates. From left to right (and top to bottom): elevation, temperature (PC), precipitation (PC), Humidity (PC), Range (Log10), temperature stability since Mid Holocene, temperature stability since Last Glacial Maximum, PC_Size/Longevity, PC_Fecundity. Source data are provided as a Source Data file.

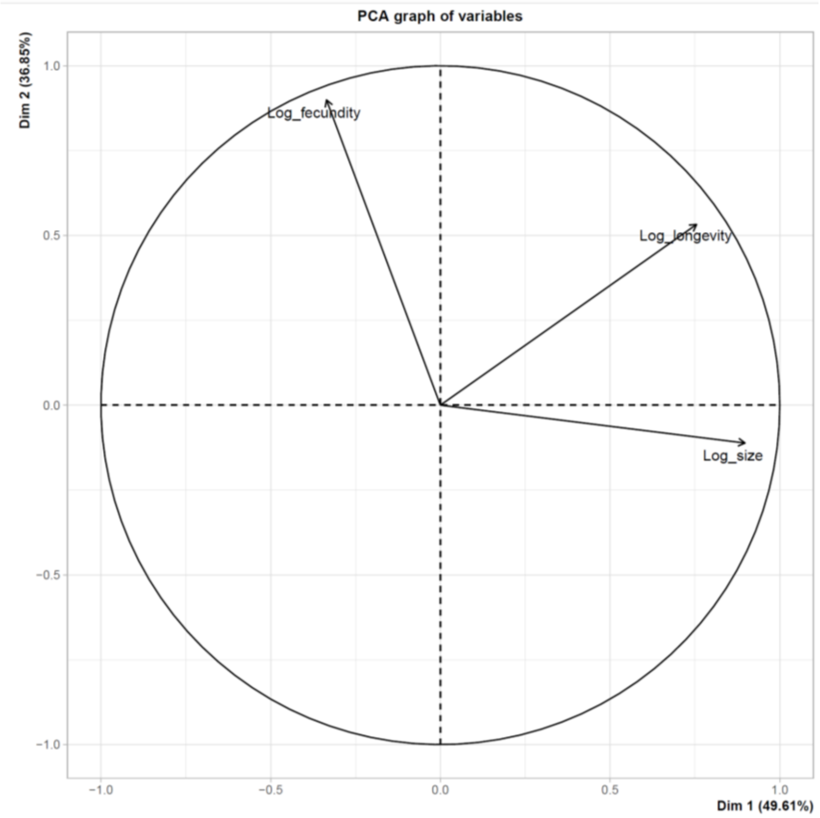

Supplementary Fig. 7. **Life history biplot.** The biplot shows contibutions of life history traits to the two principal components “PC_SizeLongevity” and “PC_Fecundity”.
