# Supplementary material for: Life history, climate and biogeography interactively affect worldwide genetic diversity of plant and animal populations
Source: Nat Commun. 2021 Jan 22;12:516. doi: 10.1038/s41467-021-20958-2 (PMC7822833; doi:10.1038/s41467-021-20958-2)
Supplement: Supplementary file 3 — Reporting Summary [file 41467_2021_20958_MOESM3_ESM.pdf]

nature research

Corresponding author(s): Hanne De Kort

Last updated by author(s): Dec 16, 2020

Reporting Summary

Nature Research wishes to improve the reproducibility of the work that we publish. This form provides structure for consistency and transparency in reporting. For further information on Nature Research policies, see our Editorial Policies and the Editorial Policy Checklist.

Statistics

For all statistical analyses, confirm that the following items are present in the figure legend, table legend, main text, or Methods section.

n/a	Confirmed	
☐	☒	The exact sample size (*n*) for each experimental group/condition, given as a discrete number and unit of measurement
☐	☒	A statement on whether measurements were taken from distinct samples or whether the same sample was measured repeatedly
☐	☒	The statistical test(s) used AND whether they are one- or two-sided<br>*Only common tests should be described solely by name; describe more complex techniques in the Methods section.*
☐	☒	A description of all covariates tested
☐	☒	A description of any assumptions or corrections, such as tests of normality and adjustment for multiple comparisons
☐	☒	A full description of the statistical parameters including central tendency (e.g. means) or other basic estimates (e.g. regression coefficient) AND variation (e.g. standard deviation) or associated estimates of uncertainty (e.g. confidence intervals)
☐	☒	For null hypothesis testing, the test statistic (e.g. F, t, r) with confidence intervals, effect sizes, degrees of freedom and P value noted<br>*Give P values as exact values whenever suitable.*
☒	☐	For Bayesian analysis, information on the choice of priors and Markov chain Monte Carlo settings
☒	☐	For hierarchical and complex designs, identification of the appropriate level for tests and full reporting of outcomes
☒	☐	Estimates of effect sizes (e.g. Cohen's d, Pearson's r), indicating how they were calculated

Our web collection on statistics for biologists contains articles on many of the points above.

Software and code

Policy information about availability of computer code

Data collection	R code was used for all analyses. Data (literature) were collected through Google Scholar. The R scripts supporting the results are available on Figshare (10.6084/m9.figshare.13373363).
Data analysis	R version 3.4.3 and R packages "glmmTMB (v1.0.2.1)", "car (v3.0-10)", "taxize (v0.9.99)", "r2glmm (v0.1.2)", "MuMIn (v1.43.17)", "vegan (v2.5-6)", and "ggplot2 (v3.3.2)" were used for data analysis and visualization.

For manuscripts utilizing custom algorithms or software that are central to the research but not yet described in published literature, software must be made available to editors and reviewers. We strongly encourage code deposition in a community repository (e.g. GitHub). See the Nature Research guidelines for submitting code & software for further information.

Data

Policy information about availability of data

All manuscripts must include a data availability statement. This statement should provide the following information, where applicable:

- Accession codes, unique identifiers, or web links for publicly available datasets
- A list of figures that have associated raw data
- A description of any restrictions on data availability

All datasets supporting the results are deposited on Figshare (10.6084/m9.figshare.13373363).

Field-specific reporting

Please select the one below that is the best fit for your research. If you are not sure, read the appropriate sections before making your selection.

- [ ] Life sciences
- [ ] Behavioural & social sciences
- [x] Ecological, evolutionary & environmental sciences

For a reference copy of the document with all sections, see nature.com/documents/nr-reporting-summary-flat.pdf

Ecological, evolutionary & environmental sciences study design

All studies must disclose on these points even when the disclosure is negative.

Study description	Meta-analysis to study the natural drivers of population genetic diversity, based on 8391 populations from 242 eudicot, 10 magnolid, 82 monocot, 50 pine, 51 amphibian, 36 reptile, 44 mollusc, 139 mammal, and 73 bird species. Sampling unit = population, nested within genera.
Research sample	Genetic diversity and geographical coordinates of all populations involved in this meta-analysis were obtained from published studies, consulted through Google Scholar. Data are provided as Supplementary Data 1, and the published studies from which these data were obtained are listed in Supplementary Data 2. Metadata describing sources of information are provided as Supplementare Data 3.
Sampling strategy	or an optimal representation of global patterns of genetic diversity, we extracted all published population genetic diversity (expected heterozygosity) data from 2000 up to 2015. We thus maximized sampling sizes.
Data collection	Using the search terms “expected heterozygosity” AND “genetic marker” AND “plant populations” OR “amphibian populations” OR “reptile populations” OR “bird populations” OR “mammal populations” OR “mollusc populations”, from 2000 up to 2015, we screened google scholar for articles that estimated population genetic diversity.
Timing and spatial scale	We extracted data from studies published between 2000 and 2015, involving research from around the globe.
Data exclusions	We only included studies that were representative for natural genetic diversity, i.e., we eliminated articles involving introduced species, invasive populations and cultivars. Studies that did not provide a map or coordinates of each population were excluded, and so were populations with sample sizes lower than 10 individuals. We focussed on terrestrial and freshwater processes and therefore excluded all marine populations.
Reproducibility	Scripts have been provided and all data were properly organized. In addition, methods were provided in a step-by-step manner. We thus have high confidence that all results obtained here can be reproduced.
Randomization	We controled for genetic relatedness and unequal sampling sizes through random effects in our models. Spatial autocorrelation was accounted for by including geographical coordinates as covariates.
Blinding	Blinding was not relevant to our study since we did not perform experimental or field work.

Did the study involve field work? [ ] Yes [x] No

Reporting for specific materials, systems and methods

We require information from authors about some types of materials, experimental systems and methods used in many studies. Here, indicate whether each material, system or method listed is relevant to your study. If you are not sure if a list item applies to your research, read the appropriate section before selecting a response.

Materials & experimental systems

n/a	Involved in the study	
[x]	[ ]	Antibodies
[x]	[ ]	Eukaryotic cell lines
[x]	[ ]	Palaeontology and archaeology
[x]	[ ]	Animals and other organisms
[x]	[ ]	Human research participants
[x]	[ ]	Clinical data
[x]	[ ]	Dual use research of concern

Methods

n/a	Involved in the study	
[x]	[ ]	ChIP-seq
[x]	[ ]	Flow cytometry
[x]	[ ]	MRI-based neuroimaging
