# Supplementary material for: Life history, climate and biogeography interactively affect worldwide genetic diversity of plant and animal populations
Source: Nat Commun. 2021 Jan 22;12:516. doi: 10.1038/s41467-021-20958-2 (PMC7822833; doi:10.1038/s41467-021-20958-2)
Supplement: Supplementary file 4 — Description of Additional Supplementary Files [file 41467_2021_20958_MOESM4_ESM.pdf]

Description of Additional Supplementary Files

Supplementary Data 1. **Dataset.** Variable values for each population, including normalized genetic diversity (GDp_norm, response variable), methodological, geographic, phylogenetic, (past) climate and life history information. Please find a detailed description of each variable in the Meta-data sheet (Supplementary Data 3).

Supplementary Data 2. **Studies**. Studies from which the expected heterzogysity and geographical coordinates were extracted.

Supplementary Data 3. **Meta-data.** Source of the information is provided where applicable ("Study" refers to the original peer-reviewed papers that were collected for this quantitative review).

Supplementary Data 4. **Kingdom Model Output.** Outcomes, explained variance (R^2m and R^2c) and model fit graphs of the desciriptive Kingdom model. The p-values of this linear mixed model are not corrected for multiple testing.

Supplementary Data 5. **Phylum Model Output**. Outcomes, explained variance (R^2m and R^2c) and model fit graphs of the desciriptive Phylum model. The p-values of this linear mixed model are not corrected for multiple testing.

Supplementary Data 6. **Animal kingdom model Output**. Outcomes, explained variance (R^2m and R^2c) and model fit graphs of the Animal Kingdom model. The p-values of this linear mixed model are not corrected for multiple testing.

Supplementary Data 7. **Plant kingdom model Output**. Outcomes, explained variance (R^2m and R^2c) and model fit graphs of the Plant kingdom model. The p-values of this linear mixed model are not corrected for multiple testing.

Supplementary Data 8**. Animal dredge table.** Dredge tables showing variable estimates (0 is = no significant contribution) for all models with ΔAIC <4 to assess variable importance (see Fig.3 in main text).

Supplementary Data 9**. Plant dredge table.** Dredge tables showing variable estimates (0 is = no significant contribution) for all models with ΔAIC <4 to assess variable importance (see Fig.3 in main text).
