# Supplementary material for: Life history, climate and biogeography interactively affect worldwide genetic diversity of plant and animal populations
Source: Nat Commun. 2021 Jan 22;12:516. doi: 10.1038/s41467-021-20958-2 (PMC7822833; doi:10.1038/s41467-021-20958-2)
Supplement: Supplementary file 2 — Peer Review File [file 41467_2021_20958_MOESM2_ESM.pdf]

Reviewers' comments:

Reviewer #1 (Remarks to the Author):

Dear authors,
Thank you for your manuscript which I enjoyed reading. It provides a global overview of the effects of different and important biological, climatic and biogeographic factors on genetic diversity, the fuel of evolution and any adaptive change and a too seldom considered aspect of biodiversity conservation and ecosystem dynamics. I found the manuscript provided important results that may explain what drives genetic diversity in natural populations and that contribute to streamlining genetic diversity as a conservation indicator.

I have several remarks:
- In the introduction, you claim to have conducted a meta-analysis, yet it is not the meta-analysis framework (calculating an effect size) that has been used here. You say in the methods that heterozygosity was chosen as an effect size, but usually an effect size is a response to a treatment or factor and it has to follow some statistical properties such as having a variance. Perhaps, the term meta-analysis or effect size should not be used here.
- I wonder if a publication effect should not have been used in the models. Data for several species may come from the same publication and thus not be independent.
- I wonder if past climate would not have been more pertinent to use than current climate. By using current climate, you conclude that it is less important in shaping genetic diversity than life history traits and biogeography. Using past climate, and how it changed, as a factor might have led to different results as past climate affected strongly population sizes and movements during contraction and colonization events. At least some caution should be shown in the conclusions.
- In the discussion section, there is a suggestion (line 277 and below) to look for "traits that may deserve more attention in future projects...". I wonder from which line of reasoning this list of traits comes from and why the traits have not been investigated in this study.
- In the discussion section, there is also a consideration of human impact (line 287 and below), concluding on a lack of evidence of human impact in the study. Testing properly for human impact would have necessitated diachronic sampling (as said in the methods, line 338). In this study, the evidence is indirect and confounded with the sampling strategy.
- I found the mention of species level genetic diversity (GDs) a bit confusing. Box 1 does not show how GDp and GDs differ in how they are measured and I do not understand the reason for lines 673-678: do you wish to show that GDp and GDs can be used as indicators of very different ecological, biological and geographical processes? Also, the terms "usually" in the box should not be used as alpha, beta and gamma diversities are seldom used terms in the population genetics literature. Identifying better what the differences are between the two measures should make the usefulness of their comparison clearer in the discussion section.

About the text itself and its structure, and the figures and tables:
- The first sentence of the introduction is a bit misleading as human mediated effects on genetic diversity are not tested (which could have been interesting). I suggest deleting it.
- The first sentences of the results are actually methods and I suggest deleting them.
- The first paragraph of the discussion is not "to the point" and should also be deleted or moved to the conclusion section.
- You talk about ecological factors when in fact you actually test for life history trait effects, thus biological factors. I suggest changing ecological for biological in many places.
- I find that Fig. 1 does not clarify the modeling framework used. And what are the differently colored dots referring to?
- What is the meaning of "n" in Supp Figure 2?
- Supplementary tables are very useful. For their full use and possible re-use, all abbreviations and what data mean in each cell should be fully explained in a metadata sheet or a supplementary

metadata file. This is usually expected from most data repositories and I advise you to choose a repository that actually asks for this.

Sincerely

Reviewer #2 (Remarks to the Author):

De Kort and colleagues provide a timely contribution to our current understanding of the global patterns of intra-specific genetic diversity across thousands of populations and species globally. The study reveals the effects of traits, climate and biogeography in the distribution of intra-specific genetic diversity. To do this, authors have conducted a meta-analysis of published literature and pull together a vast amount of data, from ecological traits to phylogenies and distributions. The study provides both confirmation of previous findings (i.e., diversity in plants is lower than in animals overall) and novel findings for a global scale (i.e., populations in the core of the range host larger genetic variability than in the periphery). These results on the core-periphery hypothesis would have the potential to be of utmost significance for conservation assessments and policy recommendations.

The study, however, faces significant challenges, some of them common to the use of meta-analysis in ecology and evolution. As Robert Whitaker for example proposed in his 2010 Ecology paper 'In the dragon's den: a response to the meta-analysis forum contributions': "…to call for great care, improved rigor, and transparency in the use of meta-analysis tools in ecology". This study by De Kort is in need of a much-improved description of the decisions, protocols, and criteria behind this meta-analysis. I doubt that, given the information provided in this study, other scientists would be able to replicate the analysis. Authors should improve the transparency and reproducibility of the study. The criteria for the selection of the datasets is not fully clear yet. For example, heterozygosity, H, can be estimated using different methods. How do we know that the estimates based on different methods across the reviewed papers are fully comparable? Or that the spatial scales at which the original populations were sampled are fully comparable (i.e, local vs landscape scale)? Or how the results may be biased by the use of different markers to estimate heterozygosity? I acknowledge that authors explore analytically some of these issues in their statistical predictive model using random effects but future readers would like to know more about how the decision taken to build the dataset may affect the results arising of the full meta-analysis approach, and also have access to the full list of studies used for the meta-analysis.

This brings me to a second significant challenge. The majority of the variance in H is explained by methodological "noise" and just a small part by abiotic or biotic processes. Authors do not hide this and indeed a full section within the discussion, which I applaud. However, and if my understanding of the results is right, the 90% of the variance approximately is explained by this noise and only a 10% by the biological relevant variables. When reading the title or the discussion, I think that authors should be even more strict on presenting this "issue". For example, would not be better to start to discussion acknowledging this and start with the "generality vs noise" section? Authors should provide the most nuanced view possible on this aspect, as in failing to do this, the paper can be heavily criticized after publication and also reduce its impact in the area of conservation biology.

Other worrying aspect is the use of longitude, latitude and altitude. Those are just human-made measures which by themselves do not control any biological mechanism or pattern. With altitude changes climate or atmospheric pressure and with latitude and longitude also climate but many other relevant biological factors. I understand the use of latitude and longitude to control for auto-correlation or to describe statistically a spatial cline but they are not bringing any light for testing abiotic and biotic drivers. Indeed, the use of latitude and longitude to test for large scale patterns of biological diversity has been abandon in Macroecology for more than a decade now.

Please, better describe the theories and predictions relating current climatic conditions to H. I wonder what effect current climatic conditions (usually estimated over a 30 year period) may have on values of H, being genetic diversity in a large part the result of dynamics in longer time-scales. Why do not use paleoclimatic conditions for example?

Overall, I find a poor reporting of the datasets across this study. For example, authors do not report what climatic sources they have use, what specific climatic variables (i.e., annual average temperature?). Report please also the units of these variables

Given the large amount of noise in the results, It also would be relevant to find in the discussion what would be the best route(s) to improve the quality, comparability, spatial and temporal coverage of population level genetic diversity; and maybe a more realistic discussion on how the findings of this study (given the constraints in data) can inform and/or improve conservation of biological diversity.

Reviewer #3 (Remarks to the Author):

In this manuscript, the authors conduct a meta-analysis of local genetic diversity, attempting to untangle a large number of hypothesized effects on it. A set of very complex analyses is undertaken, resulting in models that are almost certainly seriously over-fitted. The vast majority of the variance in these models is accounted for by the random effects, which may control out signal. The tiny effect sizes of the fixed effects are then presented as important conceptual advances in our knowledge. I expand on these criticisms below.

I do think it is worth doing a meta-analysis of local genetic diversity, so I have no problem with the core concept of the paper. Whether it is as 'crucial' (etc) as the authors repeatedly claim in the manuscript is more debatable. There seems to be a lot of hyperbole that is best deleted; the much more neutral but informative text in Box 1 is far more compelling, in my opinion.

I also suggest that factors that promote local genetic diversity (what they label GD_P) may not necessarily promote beta genetic diversity (perhaps even a negative relationship between local and beta?), such that the overall implications of the findings for conservation are more limited than the authors imply. Overall, as suggested in L89, we know quite a lot about species-level genetic diversity, and although the authors do supply some valid reasons why it is important to also know about population genetic diversity, I am not convinced that it is as important as they claim.

Overfitting. The modelling is a major issue in my opinion. The authors fitted hugely complex models (see Models 1, 2 and 3 in the Methods section), with 'all possible variants of the full model' (L388) and then selected the one with the lowest AICc. The only other models considered as alternatives were ones with delta AICc less than 2. AIC does not solve problems associated with multicollinearity or spatial autocorrelation, and for these reasons among others, the modelling approach taken in this manuscript strikes me as being as close to guaranteeing over-fitting as one can get. (I find it ironic that the authors say that this approach is 'for the sake of simplicity', L389!) I am almost certain that the models contain considerable artefact. Various of the diagnostics reinforce this impression for me, including the strong kurtosis of the residuals and the near-zero error variance.

To exemplify, this is the 'best' model (i.e. after simplification) for the taxonomic modelling, according to Supp Table 4:
'Best_model = lmer(GDp ~ |Latitude| + Altitude + Position + Temperature + Precipitation + Humidity + Phylum*Temperature + Phylum*Precipitation + Phylum*Humidity + Phylum*Altitude + Phylum + (1 | Genus/Species) + (1 | marker/loci) + (1 | SampleSize), data = datsc, REML = FALSE)'

Tiny effect sizes. Within all that complexity, the overwhelming majority (more than 90%) of the

explanatory power was in the random effects (L131), leaving very small effect sizes (totalling 9%) for the fixed effects, which are the core results of the manuscript. The explanatory power of the strongest fixed effects that is then reported in the next few lines (e.g. 32% for phylum, 25% for phylum:latitude interaction) is misleading because these are percentages of 9%, actually meaning 3% and 2% of the overall variation respectively, in these two cases. Those examples are from the taxonomic analysis; the equivalent numbers for the other two analyses are nearly identical. Fig 2 is thus also highly misleading.

Could the low explanatory power of the fixed effects be to do with always including 'genus/species' in the random effects? If I have understood this right, this may a priori remove most meaningful signal from life history variables like dispersal type and pollen vector type, and probably other fixed effects too. (Note: L197 seems to reinforce my suspicion that this is a problem in the analysis.) This probably seriously distorted the main findings of the manuscript.

Here is another aspect of the analysis that does not fill me with confidence. From L353:
'we calculated orthogonal polynomials from the latitudes using the function 'poly' from the R package Stats, rendering two statistically independent latitudinal variables: the first one (hereafter "Latitude") was correlated with the original latitudinal data, and the second one (hereafter "|Latitude|") with the distance to the Equator. While "Latitude" and "Longitude" were included to account for spatial autocorrelation, "|Latitude|" accounted for variation in ecological and environmental conditions, especially through its association with temperature, photoperiod and historical range expansions.'
If I have understood correctly, the absolute latitude variable is simply the square of the latitude. Given that latitude has both negative (southern hemisphere) and positive (N. hemis) values, while the overall linear correlation between the two variables is close to zero, calling the two variables 'statistically independent' is nonsense. This is well illustrated by the top-left graph in the Supp Methods 'Figure', which shows a perfect non-linear relationship.

SPECIFICS – selected

The Methods section and Supp Fig 2 implies that the search terms for finding datasets all included only the plural 'populations' as exact terms, and not the singular 'population'. Being within double quotations presumably only found exact matches, thus potentially missing a lot of relevant literature. If this suspicion is true then it is a serious issue for this manuscript.

There are many problems with the level of explanation and presentation of the manuscript, and I spent much longer than I should have needed to, trying to figure out what was done and what the results are showing. I urge the authors to have more respect for reviewers' time. We should not have to spend ages guessing what things are, trying to find explanations, etc. Here is just a selection of the problems:

The literature search excluded 'pages > 50' (Supp Fig 2) but this is not explained.

Readability is reduced by use of abbreviations in the text (e.g. Ne, H_E, GD_P, GD_S).

There are some problems with the referencing (a few author-date, not numbered; some of the numbering is wrong). E.g. L65, L92.

L166 refers to a Fig65B!

In Fig 1b I cannot tell which phylum is which, nor what the units are on the axes.

Fig.2 caption mentions red and green colours - bad practice for inclusivity! I actually could not see any colours in this figure until I zoomed in to 350%, and even then found it difficult to distinguish them. It was only at 500% that I could distinguish in Fig 4. In these cases it matters little because I know

which taxa are plants and which are animals, but Supp Methods funnel plot figure (unlabelled - at the end of the document) is a nightmare, especially for a red-green colour-blind person like me!

Supp Fig 1 is very poorly explained.

The Supp Tables need proper explanation. For example, there are three latitude columns (Latitude, latitude and abs(latitude)) in Supp Table 1; it needs to be explained that one is ‘raw’, one is linearly rescaled and the other is the ‘absolute’ latitude variable, which is just the square of latitude (then rescaled, I guess). This Table refers to the supplementary methods for explanation, but the three latitude variables are not explained there. Users should not have to guess these things.

Supp Methods residual plots: various problems. For example, Fig A is one graph but its caption says there is a left panel and a right panel. Fig B has 5 panels, not reflected in the Supp Tables. Etc. Again, far too little explanation.

**Reviewer #1**

*Thank you for your manuscript which I enjoyed reading. It provides a global overview of the effects of different and important biological, climatic and biogeographic factors on genetic diversity, the fuel of evolution and any adaptive change and a too seldom considered aspect of biodiversity conservation and ecosystem dynamics. I found the manuscript provided important results that may explain what drives genetic diversity in natural populations and that contribute to streamlining genetic diversity as a conservation indicator.*

**Reply**: We thank the referee for the appreciation. We hope to trigger similar enthusiasm at the readership front. Please find below our feedback to each of the comments.

*I have several remarks:*
**2.** *In the introduction, you claim to have conducted a meta-analysis, yet it is not the meta-analysis framework (calculating an effect size) that has been used here. You say in the methods that heterozygosity was chosen as an effect size, but usually an effect size is a response to a treatment or factor and it has to follow some statistical properties such as having a variance. Perhaps, the term meta-analysis or effect size should not be used here.*

**Reply**: We agree, and now refer to "quantitative review" and "study" instead of "meta-analysis", and to "response variable" instead of "effect size" throughout the text. Note however that for modeling, we used some approaches classically used in meta-analyses, such as the inclusion of a weighting parameter to give more weight to studies with more accurate genetic diversity estimates (i.e. those with larger sample size and higher number of loci).

**3.** *I wonder if a **publication effect** should not have been used in the models. Data for several species may come from the same publication and thus not be independent.*

**Reply**: We actually considered including a study effect as a random variable, but realizing that the "species" effect and the "study" effect are highly congruent, we feared that this would unnecessarily complicate the random structure of the model. To show that the "species" and "study" effect render similar outcomes, we re-ran our final models with "study" effect instead of "species" effect. For most models, the results confirm that, when "study" was added as a random factor accounting for non-independence due to publication-related methodological choices and study-related spatial scale of sampling (See also **comment 23**), results are nearly identical. The Animal kingdom model failed to converge, however, when replacing "species" by "study", probably because of the more fractionate random structure when using "study" (there are more studies than species). We conclude that accounting for the "species" effect is the most appropriate to account for issues of non-independency. Please evaluate in the table below the overall results of the model alternatives in terms of significant variables and explained variance.

| Model | Significant when "Species" random | Significant when "Study" random | $R^2m/R^2c$ ("Species") | $R^2m/R^2c$ ("Study") |
|---|---|---|---|---|
| **Kingdom** | Kingdom*Latitude | Kingdom*Latitude | 0.08/0.76 | 0.07/0.78 |
| **Phylum** | Phylum*Latitude | Phylum*Latitude | 0.11/0.77 | 0.10/0.78 |
| **Animal kingdom** | Prec,Temp,Longevity,Range*Phylum, Range*Position,Fecundity*Position, MH | No convergence | 0.32/0.71 | NA |

| | | | | |
|---|---|---|---|---|
| | stability*Phylum, LGM stability*Phylum | | | |
| **Plant kingdom** | Prec,Lifeform,Seed*Position | Prec,Lifeform,Seed*Position | 0.10/0.79 | 0.10/0.83 |

**4.** *I wonder if* ***past climate*** *would not have been more pertinent to use than current climate. By using current climate, you conclude that it is less important in shaping genetic diversity than life history traits and biogeography. Using past climate, and how it changed, as a factor might have led to different results as past climate affected strongly population sizes and movements during contraction and colonization events. At least some caution should be shown in the conclusions.*

**Reply**: This is a very interesting and valid point. We now included paleoclimate (last glacial maximum LGM and mid-Holocene MH) in the models (see also **comment 18**). Because paleoclimate and current climate are extremely correlated, we focused on temperature stability since LGM and MHC, hypothesizing that (i) a more stable climate results in higher $GD_P$, and (ii) past climate variability is more important than current climate in driving global $GD_P$. Interestingly, some but not all phyla showed the expected positive correlation between past temperature stability and $GD_P$. Mammals showed the strongest correlation between $GD_P$ and temperature stability since the LGM, while amphibians and molluscs showed the strongest correlation between $GD_P$ and temperature stability since the MH (New Fig. 4). We suspect dispersal to play an important role in the rate and timing of post-glacial re-colonization, as molluscs and amphibians (poor dispersal) vs. mammals (high dispersal) left $GD_P$ signatures of relatively recent (since Mid-Holocene ca. 6000 years ago) vs. ancient (since LGM ca. 22000 years ago) population expansion, respectively. Please find below the included elaborate discussion focusing on these new results.

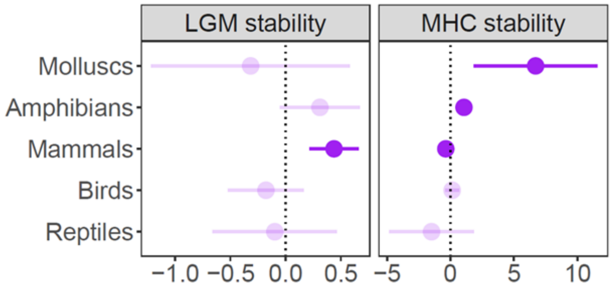


**New Fig. 4** (limited to the stability effects). Effects of LGM and MH stability on $GD_P$. Non-significant effects (p-values > 0.05) are transparent.

**New L2787-299 (discussion)**: "The negative relationship between population genetic diversity and latitude in amphibians and molluscs conforms with the positive relationship between their population genetic diversity and **temperature stability** since the Mid-Holocene (Fig 4D), which provides support for the hypothesis that latitudinal gradients of population genetic diversity likely result from longer term population persistence associated with more stable climates in the past[43,64]. Through hampering post-glacial movement, poor dispersal abilities (range effect in Fig. 4D) in particular may play a critical role in driving effective population size in amphibians and molluscs in response to past climatic conditions [49,65–69] (Fig. 4D). As the only vertebrate group with a positive relationship between LGM temperature stability and population genetic diversity, mammals seem to manifest the longest-lasting imprint of temperature stability on population genetic diversity. This

result suggests that mammals exerted relatively rapid post-glacial re-colonization, explaining why mammal population genetic diversity coincides with LGM rather than with MH climate stability."

**5.** *In the discussion section, there is a suggestion (line 277 and below) to look for "traits that may deserve more attention in future projects...". I wonder from which line of reasoning this list of traits comes from and why the traits have not been investigated in this study.*

**Reply**: We added some more references to justify the potential effect of these traits on $GD_P$. We did not explicitly test the impact of these traits on $GD_P$ because they are hard to obtain reliably for many species in our dataset. And perhaps more importantly, adding more traits to the models would make them even more complex (see comment **23** from referee 3 for issues regarding model complexity). We thus focused on those traits with highest data availability but also with the strongest hypothesized effects on global $GD_P$ patterns.

**New L428-432 (discussion)**: "*These traits are, however, currently poorly available for a large number of species, preventing us from including them in our models. The exploration of anthropogenic impacts on population genetic diversity may finally uncover additional global patterns, allowing accounting for anthropogenic effects while establishing natural baseline population genetic diversity values*."

**Added references**:

Álvarez-Noriega, M. *et al.* Global biogeography of marine dispersal potential. *Nat. Ecol. Evol.* 1–8 (2020). doi:10.1038/s41559-020-1238-y.

Ayre, D., O'Brien, E., Ottewell, K. & Whelan, R. The accumulation of genetic diversity within a canopy-stored seed bank. *Mol. Ecol.* **19**, 2640–2650 (2010).

Tellier, A. Persistent seed banking as eco-evolutionary determinant of plant nucleotide diversity: novel population genetics insights. *New Phytol.* **221**, 725–730 (2019).

**6**. *In the discussion section, there is also a consideration of human impact (line 287 and below), concluding on a lack of evidence of human impact in the study. Testing properly for human impact would have necessitated diachronic sampling (as said in the methods, line 338). In this study, the evidence is indirect and confounded with the sampling strategy.*

**Reply**: We fully agree and thank the referee for this important remark. We made two major changes to address this issue. First, we removed our test of human impact because we did not sample diachronically, as indicated by the referee. Second, we added a new categorical life history variable to the plant dataset (Lifeform classified into "annual", "perennial herb", "shrub" and "tree"), because long-living tree species have been previously found to be susceptible to an anthropogenic extinction debt (e.g. Vranckx et al. 2012). If human impacts such as habitat fragmentation have considerably impacted plant populations across the globe, which is not unreasonable, we expect lower $GD_P$ for annuals than for long-living shrubs and trees. We correspondingly found that long-living plants such as shrubs and trees harbor consistently higher $GD_P$ than short-living plants, indeed suggesting that long-living plants suffer from an extinction debt caused by habitat fragmentation. We now added some lines to acknowledge this concern.

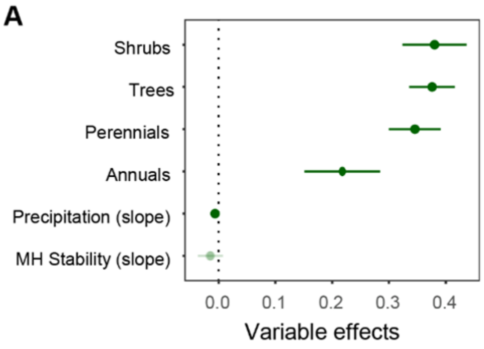


**New Fig. 5A**. Variable effects on plant $GD_P$. Non-significant effects (p-values > 0.05) are transparent.

**New L359-366 (discussion):** "*Our finding that plant* ***life-form*** *is associated with population genetic diversity likely reflects the presumable impact of habitat fragmentation and reconfiguration on global patterns of* $GD_P$*, since long-living plants respond more slowly to reduced gene flow. This extinction debt, predominantly linked to woody species, has been of considerable concern to nature conservation, and indicates an underestimation of the number of long-living species endangered by habitat fragmentation*[82–85]*. Although we did not specifically collect studies in the context of habitat fragmentation, land-use change and consequent fragmentation are among the most pronounced global change drivers across the earth, and have most likely affected most populations to some extent*[86,87]*.*"

**7.** *I found the mention of species level genetic diversity (GDs) a bit confusing. Box 1 does not show how GDp and GDs differ in how they are measured and I do not understand the reason for lines 673-678: do you wish to show that GDp and GDs can be used as indicators of very different ecological, biological and geographical processes? Also, the terms "usually" in the box should not be used as alpha, beta and gamma diversities are seldom used terms in the population genetics literature. Identifying better what the differences are between the two measures should make the usefulness of their comparison clearer in the discussion section.*

__**Reply**__: We agree that a clearer description of both parameters was required to differentiate and allow comparison of our $\mathbf{GD_P}$ results, with recently published $\mathbf{GD_S}$ studies. Both metrics are measured by shared methodologies, but are based on a different sample composition. $GD_S$ is calculated from a number of unrelated individuals across the species range. Because these individuals are sampled from distinct populations that evolved independently, $GD_S$ does not capture the effects of the local environmental and the biogeographic context on the extant genetic diversity within individual populations. $GD_P$, on the other hand, is calculated from individuals from the same population which share the same eco-evolutionary history. As opposed to $GD_S$, $GD_P$ is thus shaped by local environmental settings such as those related to current and past climate, biogeography and habitat configuration. As a consequence, $GD_P$, but not $GD_S$, represents the genetic variability and evolutionary potential of a population, with low $GD_P$ typically representing increased levels of inbreeding and decreased potential to evolve. While similar eco-evolutionary and biological processes may drive $GD_P$ and $GD_S$, the outcome of these processes on $GD_P$ and $GD_S$ can be very different. For example, at the edge of a species distribution, $GD_P$ is expected to be low due to low connectivity to other populations and thus reduced gene flow. The same biogeographic context (i.e. edge of a species distribution), however, may increase $GD_S$ across individuals sampled from different populations because the individuals originating from the distribution edge may harbor a genetic

signature that is very different from that of the other individuals that are included to calculate $GD_S$. We now added these clarifications to the box. As requested, we also removed the inappropriate term “usually” from the box.

**New L57-61** (**Introduction**): “*Population genetic diversity ($GD_P$) is a key indicator of population fitness and evolutionary potential[6,7] that describes the genetic diversity of local populations. As a consequence, $GD_P$ is often used as a key estimate to identify “evolutionary significant units” (ESUs, Box 1), upon which conservation programs frequently rely to inform about the evolutionary and demographic history of populations[8,9].*”

**New L836-859 (Box 1)**: “*All differences between $GD_P$ (local scale) and $GD_S$ (range-wide scale) can be reduced to the sampling scale, i.e. the scale at which individuals were sampled to calculate GD. Specifically, $GD_S$ does not capture the effects of the local environmental and biogeographic context on individual populations because the sampled individuals originate from distinct populations that evolved independently. $GD_P$, on the other hand, is calculated for a population of individuals sharing the same eco-evolutionary history. Therefore, $GD_P$ is commonly used in population genetic studies as it captures the effects of the local environment and more contemporary processes (mutation, gene flow, drift and selection), as well as genetic signatures of events in the more distant past. Distinct local populations, each featured by a particular $GD_P$ value, are also referred to as evolutionary significant units (ESUs), i.e. distinct population units that require separate management because they experienced independent evolutionary histories[8].*

*While similar eco-evolutionary and biological processes may drive both $GD_P$ and $GD_S$, the outcome of these processes on $GD_P$ and $GD_S$ can be very different. For example, at the periphery of a species distribution, $GD_P$ is expected to be low due to low connectivity to other populations and thus reduced gene flow. The same biogeographic context (i.e. at the distribution edge), however, may increase $GD_S$ across individuals sampled in independent edge populations because these populations have distinct genetic signatures.*

*Most species are facing population declines on local and regional scales, and can benefit considerably from local conservation efforts that can prevent considerably population extinctions. Therefore, a $GD_P$ baseline (which defines how genetic diversity is predicted to vary according to the environmental and biogeographic properties of populations within and across species) to which conservation practitioners may tailor their conservation strategy, could facilitate local population restoration before extinctions and, eventually, major range contractions occur.*”

**8.** *About the text itself and its structure, and the figures and tables:*

*The first sentence of the introduction is a bit misleading as human mediated effects on genetic diversity are not tested (which could have been interesting). I suggest deleting it.*

**Reply**: We agree and removed this sentence.

**9**. *The first sentences of the results are actually methods and I suggest deleting them.*

**Reply**: Done.

**10**. *The first paragraph of the discussion is not "to the point" and should also be deleted or moved to the conclusion section.*

**Reply**: As suggested, we integrated this paragraph in the conclusion section, and wrote a new first paragraph that is more "to the point" to outline the discussion. If you feel that this adjusted first paragraph is also unnecessary, we are of course prepared to remove it.

**New L228-236: "***Our study provides the first attempt to reveal worldwide patterns and underlying drivers of genetic diversity at the local population scale across a broad range of animal and plants species. We first discuss the broad patterns of plant and animal population genetic diversity, as well as the only general pattern that seem to hold true for both plants and animals. Specifically, we found that core populations systematically harbour higher genetic diversity than edge populations. While this general, phylum-independent pattern is relatively subtle, the phylum-specific impacts of temperature stability, life history traits, and biogeographical position are more pronounced and will be discussed for plants and animals separately. We finally provide conservation implications, in addition to limits and prospects for future studies*."

**New L434-452 (conclusion)**: "*Comprehensively considering the wide array of factors that can simultaneously and interactively affect genetic diversity at the local (population) scale is crucial for understanding the processes affecting evolution and adaptability of species. Here, we provide, for the first time, a global map of within-population genetic diversity, and we demonstrate surprisingly distinct patterns and drivers of population genetic diversity between plants and animals. While contemporary climate, biogeography and life history traits all affect both plant and animal genetic diversity, the direction and strength of these relationships substantially differ between and within kingdoms, except for one general law (i.e. the core-periphery hypothesis) that applies to all plant and animal phyla. We further show that patterns of genetic diversity at the population scale (linked to effective population size) do not align with those typically identified for genetic diversity measure at the species level (linked to diversification rates), and that genetic diversity at the population scale is shaped by the interplay between historical climate, current climate, biogeography and life history. This interplay questions several conventional assumptions, such as the expectation that island populations and endemic species systematically harbour low levels of genetic diversity. Our findings have major conservation implications, and raise questions regarding the efficacy of formulating management solely based on genetic diversity measured at the species level or on range-wide measures of population size. Instead, our results encourage the development a conservation framework based on genetic diversity measured at the population scale and accounting for biogeographic context and life history traits*."

**11.** *You talk about ecological factors when in fact you actually test for life history trait effects, thus biological factors. I suggest changing ecological for biological in many places.*

**Reply**: We replaced "ecological factors" by "biological factors" where appropriate (e.g. **New L29**, **New L49** and **New L125**).

**12**. *I find that Fig. 1 does not clarify the modeling framework used. And what are the differently colored dots referring to?*

**Reply**: Because Fig. 1 (the modeling framework) was confusing, we removed it and instead were more clear in the methods. The second panel, describing the life history traits, is now treated as a supporting figure. We now clarified the colors in the caption.

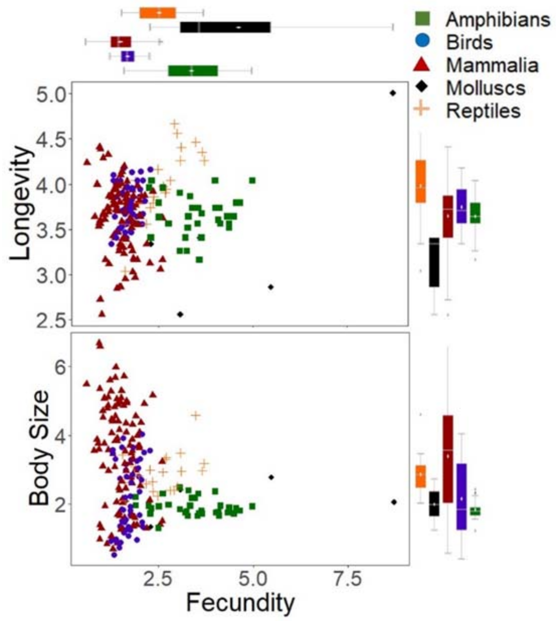


**New Fig. S2.** Distribution of life history traits (all log10-transformed) across phyla. The black outlier at the right is the freshwater pearl mussel *Margaritifera margaritifera*, featured by small body size, high fecundity rates and a life span exceeding 200 years. Box plots have same units as corresponding scatter plot axis: (Longevity = log10(days); Body Size = log10(cm); Fecundity = log10(lifetime offspring). See Supplementary methods for a detailed description of each life history trait.

**13**. *What is the meaning of "n" in Supp Figure 2?*

**Reply**: It represented the number of publications for a specific taxon. We now added the explanation to the caption (Now Fig. S1).

**14**. *Supplementary tables are very useful. For their full use and possible re-use, all abbreviations and what data mean in each cell should be fully explained in a metadata sheet or a supplementary metadata file. This is usually expected from most data repositories and I advise you to choose a repository that actually asks for this.*

**Reply**: We now added a metadata sheet (Table S3). Upon publication, we will also make all data publically available through DRYAD (please see the “Data accessibility” section).

**Reviewer #2**

*De Kort and colleagues provide a timely contribution to our current understanding of the global patterns of intra-specific genetic diversity across thousands of populations and species globally. The study reveals the effects of traits, climate and biogeography in the distribution of intra-specific genetic diversity. To do this, authors have conducted a meta-analysis of published literature and pull together a vast amount of data, from ecological traits to phylogenies and distributions. The study provides both confirmation of previous findings (i.e., diversity in plants is lower than in animals overall) and novel findings for a global scale (i.e., populations in the core of the range host larger genetic variability than in the periphery). These results on the core-periphery hypothesis would have the potential to be of utmost significance for conservation assessments and policy recommendations.*

**15.** *The study, however, faces significant challenges, some of them common to the use of meta-analysis in ecology and evolution. As Robert Whitaker for example proposed in his 2010 Ecology paper 'In the dragon's den: a response to the meta-analysis forum contributions': "…to call for great care, improved rigor, and transparency in the use of meta-analysis tools in ecology". This study by De Kort is in need of a much-improved description of the decisions, protocols, and criteria behind this meta-analysis. I doubt that, given the information provided in this study, other scientists would be able to replicate the analysis. Authors should* ***improve the transparency and reproducibility of the study****. The criteria for the selection of the datasets is not fully clear yet. For example,* ***heterozygosity****, H, can be estimated using* ***different methods****. How do we know that the estimates based on different methods across the reviewed papers are fully comparable? Or that the* ***spatial scales*** *at which the original populations were sampled are fully comparable (i.e, local vs landscape scale)? Or how the results may be biased by the use of* ***different markers*** *to estimate heterozygosity? I acknowledge that authors explore analytically some of these issues in their statistical predictive model using random effects but future readers would like to know more about how the decision taken to build the dataset may affect the results arising of the full meta-analysis approach, and also have access to the* ***full list of studies*** *used for the meta-analysis.*

**Reply**: We agree that the transparency and reproducibility of our work deserved more attention, and we now carefully addressed each of the issues. First, we assumed that **heterozygosity** was measured following Nei's definition of gene diversity, which is sensitive to unequal sample sizes. To account for a potential bias due to unequal sample sizes, we integrated sample size in our model through a weighing factor (see below) to ensure that any variability in our data caused by different $GD_P$ calculation methodologies is accounted for. Second, we acknowledged that the spatial sampling scale as well as marker type and variability differed between studies, but that these methodological issues are accounted for in the random part of our models. Our random factor "species" captures part of this issue. Because for most species, all populations were part of the same study (and thus the same **methodology for calculation of $GD_P$**), among-study methodological differences should be captured by our "species" effect. This also includes different **spatial scales** at which the original populations were sampled. Indeed, some studies sampled their populations at very local scale while in other studies, populations were sampled at much larger landscape scales. Our "species" variable should account for varying choices of sampling across studies. Note also that, in response to referee 1 (please see **comment 3**), we ran alternative models using a "study" variable instead of a "species" variable. Both models rendered very similar outcomes, and we are confident that they both capture the methodological differences among studies. We already included "**marker**" to correct $GD_P$ for marker effects and in the previous manuscript, this explained much of the variation in $GD_P$. However, we previously did not re-scale the number of loci within marker types, which may have strongly

imbalanced the random part of our model. For example, typically much more SNPs and AFLPs (on average 1947 loci) are used than microsatellites and enzymes (on average 16 and 13, resp). We now standardized the response variable ($GD_P$) per marker type to improve the comparability between marker systems. Despite these changes made to our model, **the conclusions of our models remained unchanged**, indicating the robustness of our modeling framework. Specifically, (i) plants have systematically lower $GD_P$ than animals, and (ii) biogeography and life history are major drivers of $GD_P$. Note, however, that we were able to **increase the $R^2_m$ of our models (from 0.09 to 0.32 for the animal kingdom model, and from 0.05 to 0.10 for the plant kingdom model**) due to the inclusion of new life history and climatic variables. Please see comments below for more details about these changes. As rightfully requested, we finally provided a full **list of the studies** used in our quantitative review (see **supplementary table S2**).

**New L467-470. "***Because* $GD_P$ *is very sensitive to marker type (e.g.* $GD_P$ *is restricted between 0 and 0.5 in AFLP markers and between 0 and 1 in microsatellite markers),* $GD_P$ *values from each marker type were standardized (mean = 0 and variance = 1) to make them comparable across studies. Standardized* $GD_P$ *values were then normalized as (*$GD_P$ *_scaled-min)/(max-min) to range from 0 to 1."*

**New L525-533**: "In all models, we (i) weighted model residuals by the number of markers and sample sizes through a frequently used weighing factor in meta-regressions[110] and allowing to take into account the precision of the estimate (more weight is given to estimates with a higher precision, i.e., with higher sample size and number of loci)

$$1/\sqrt{\log(Loci\ \times SampleSize)}$$

, (ii) included "**Marker**" (*co-dominant markers* vs. *dominant markers* vs. *enzymes*) to control for non-independence within marker types, and (iii) incorporated "**Species**" (extracted using the R package *Taxize*) to control for non-independence due to both phylogenetic relatedness and study-specific methodological aspects such as $H_E$ estimation methods and sampling protocols."

16. *This brings me to a second significant challenge. The **majority of the variance in H is explained by methodological "noise"** and just a small part by abiotic or biotic processes. Authors do not hide this and indeed a full section within the discussion, which I applaud. However, and if my understanding of the results is right, the 90% of the variance approximately is explained by this noise and **only a 10% by the biological relevant variables**. When reading the title or the discussion, I think that authors should be even more strict on presenting this "issue". For example, would not be better to **start to discussion acknowledging this** and start with the "generality vs noise" section? Authors should provide the most nuanced view possible on this aspect, as in failing to do this, the paper can be heavily criticized after publication and also reduce its impact in the area of conservation biology.*

<u>**Reply**</u>: As suggested, we now moved the section on methodological noise to the first section of the discussion (the section "**General patterns of population genetic diversity", L273-282**), to make sure that the remaining discussion is interpreted in light of this methodological noise. We also refer to **comment 20** for further considerations regarding the noise issue. Note, however, that the amount of variance explained by our fixed variables has considerably increased as compared to the previous version of our manuscript (please see **comments 15** and **23**).

**17.** *Other worrying aspect is the use of* ***longitude, latitude and altitude****. Those are just human-made measures which by themselves do not control any biological mechanism or pattern. With altitude changes climate or atmospheric pressure and with latitude and longitude also climate but many other relevant biological factors. I understand the use of latitude and longitude to control for auto-correlation or to describe statistically a spatial cline but they are not bringing any light for testing abiotic and biotic drivers. Indeed,* ***the use of latitude and longitude to test for large scale patterns of biological diversity has been abandon in Macroecology for more than a decade now.***

**Reply**: We agree with the referee that latitude and longitude do not have any clear biological meaning. We therefore omitted these variables from most of the modeling (except for an explorative analysis aiming to map $GD_P$ across space and kingdoms). Instead, we (i) explored spatial patterns of $GD_P$ across taxa and space through the more biologically meaningful "distance to equator" (i.e. absolute latitude), and (ii) included variables related to historical climate stability. We kept altitude in our analyses because altitude is frequently associated with climate and/or biogeography and can explain fine-scale variation in $GD_P$. Our results confirm that there is a non-negligible altitude effect in the animal kingdom, where $GD_P$ decreases with altitude in line with the core-periphery hypothesis (populations more isolated towards the altitudinal edges of their distributions).

**New L536-541 (Methods)**: *"In a first, descriptive step, we aimed to uncover whether plant and animal species have distinct levels of $GD_P$ across the globe. Because distance to equator is thought to be an important moderator of genetic diversity through its association with temperature, photoperiod and historical range expansions, we implemented a linear mixed modelling (LMM) approach testing the impacts of |Latitude|*Kingdom while controlling for non-independence within marker types and species (see above)."*

**New L490-497**: "*In addition, because historical climate variability may have imprinted $GD_P$ (populations may have persisted much longer in regions featured by stable climates as opposed to more variable climates where population turn-over and bottlenecks are more frequent), we used averaged temperatures for the Mid Holocene climate (MH; the last 6000 years from now) and for the Last Glacial Maximum climate (LGM, the last 22000 years from now), and calculated "**MH stability**" and "**LGM stability**" as the standardized differences between the current temperature and the past temperature calculated either from LGM or MH respectively (see Supplementary Methods)."*

**New L502-506:** *""**Altitude**" was also retrieved for each population. Although altitude encompasses climatic variation among populations, climatic variation is accounted for by the three climatic variables described above. Remaining altitude effects are therefore considered as a proxy for the isolation of populations; the highest the altitude, the more isolated the population. We expected $GD_P$ to be lower at higher altitude due to an increase in spatial isolation (i.e. decrease in gene flow)."*

**New L303-306 (Discussion)**: *"Independent from these climatic effects, we found a weak, but expected, negative relation between population genetic diversity and altitude, suggesting that populations are more isolated at higher altitude. This finding is in line with the core-periphery effect that was most pronounced in the animal kingdom (Fig. 4B*)."

**18.** *Please, better describe the theories and predictions relating current climatic conditions to H. I wonder what effect current climatic conditions (usually estimated over a 30 year period) may have on values of H, being genetic diversity in a large part the result of dynamics in longer time-scales. Why do not use* ***paleoclimatic*** *conditions for example?*

**Reply**: We found this an interesting suggestion (see also **comment 4**), and now included paleoclimatic data in the analysis. Because paleoclimate and current climate are extremely correlated, we focused on climate stability since LGM and MHC, hypothesizing that (i) a more stable climate results in higher $GD_P$, (ii) long-living species manifest a stronger signal of past climate change than short-living species, and (iii) past climate variability is more important than current climate in driving global $GD_P$. Interestingly, population dynamics across longer time scales did affect global patterns of $GD_P$, but these effects were highly phylum-dependent. Please see below the specific results and discussion.

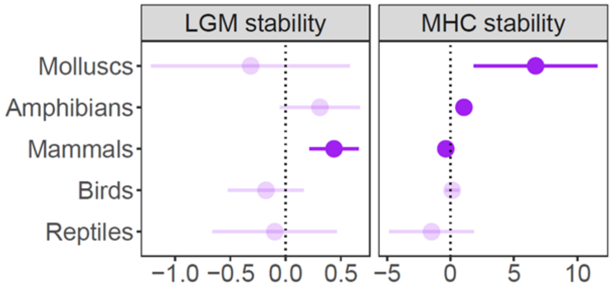


**New Fig. 4** (limited to the stability effects). Effects of LGM and MH stability on $GD_P$. Non-significant effects (p-values > 0.05) are transparent.

**New L181-185 (Results)**: "*As opposed to these general, phyla-independent effects, effects of temperature stability and species range on $GD_P$ were heterogeneous across phyla (Fig. 4D). Specifically, there was a significant increase in $GD_P$ with increasing long-term temperature stability in amphibians and molluscs since the Mid Holocene (Fig. 4D), and in mammals since the LGM (Fig. 4C).*"

**New L209-211**: "*Temperature stability since the Mid Holocene marginally and negatively influenced plant $GD_p$, but this effect was less important than current climate (precipitation) (Fig. 5A, Table S7).*"

**New L249-262 (Discussion)**: "*Our results provide surprisingly weak support for the frequently hypothesized relationship between **latitude** (distance to equator**)** and genetic diversity*[31,45,46,49,51]*. Overall, there was no latitudinal gradient (using absolute values of latitude) of population genetic diversity in plants, and a weak gradient in animals. Similarly, and contrary to the expectation that stable climates at low latitude result in high population genetic diversity due to long-term population persistence, we found poor evidence that signature of past temperature stability favours population genetic diversity (Figs 4A and 5A, but see the next paragraph for phylum-specific patterns). This may suggest (i) that contemporary processes are more important than post-glacial recolonization dynamics in explaining population genetic diversity and/or (ii) that microrefugia that are uncoupled from general macroclimatic clines contribute further than macrorefugia to past population dynamics*[60]*. Both processes likely contribute to the global population genetic diversity distribution, as (i) both contemporary climates (temperature for animals, precipitation for animals and plants, see the next sections) and biogeographical position (animals and plants) affected population genetic diversity, and (ii) a mosaic of fine-grained and large-scale population genetic diversity patterns were observed (Table S8), which is consistent with the complex spatial nature of microrefugia. Importantly, our results are in line with a recent study that could not find clear latitudinal patterns in population genetic diversity for 600 vertebrate species*[61]*. The strong discrepancy between studies assessing*

*latitudinal gradients in genetic diversity ([61 and our study vs. 45,49,51]) calls for a new paradigm regarding the worldwide distribution of population genetic diversity."*

**19**. *Overall, I find a poor reporting of the datasets across this study. For example, authors do not report what climatic sources they have use, what specific climatic variables (i.e., annual average temperature?). Report please also the units of these variables.*

**Reply**: This information was already available in the Supplementary Methods, under "environmental variables". We now also added both current climate and paleoclimate data to the Supplementary Tables (**Supp. Table S1**). Other improvements regarding data collection and reporting (e.g. addition of a metadata sheet) can be found under **comment 15**.

**20.** *Given the large amount of noise in the results, It also would be relevant to find in the discussion what would be* ***the best route(s) to improve the quality, comparability, spatial and temporal coverage of population level genetic diversity****; and maybe a more realistic discussion on* ***how the findings of this study (given the constraints in data) can inform and/or improve conservation of biological diversity.***

**Reply**: We agree that such a discussion could considerably help guiding the scientific community to improve comparability and usefulness of published population genetic diversity data. We thus added a few paragraphs to address these issues.

**New L412-422 (Discussion)**: "*The amount of variance explained by molecular marker and study effects (captured by our "species" variable) demonstrate that a more standardized methodology is crucial for allowing comparison between species and studies. The enormous discrepancy in population genetic diversity estimates among studies questions their use in biodiversity conservation, and compromises the development and operationalization of a unified population genetic diversity framework for monitoring global biodiversity (Mace et al. 2018; Jetz et al. 2019). The ongoing transformation from population genetics into population genomics is promising, since individual outlier SNPs typically have a much more reduced impact on population genetic diversity estimates than individual microsatellite loci with suspicious allele distributions. To further ensure the comparability and usefulness of published population genetic diversity metrics, a detailed description of the study species and of the geographical position of the sampled populations helps contextualizing and comparing $GD_P$ estimates."*

**New L390-395**: "O*ur findings demonstrate great potential for harmonizing $GD_P$ across space and species for generating a unified conservation genomic framework for biodiversity monitoring and prioritization. Such a universal perspective on spatial and cross-taxon $GD_P$ patterns becomes particularly appealing with the increasing use of SNPs to calculate $GD_P$, and opens the door for exploring $GD_P$-extinction risk associations across taxa to support genetic marker-based conservation assessment.*"

**New L380-389**: "*Our results illustrate that population genetic diversity ($GD_P$) and genetic diversity at the species level ($GD_S$) can have strongly different spatial patterns, likely as a result of the interplay between biogeography, climate and species traits together shaping local effective population size. This result has strong implications for the management of local populations with distinct evolutionary histories (cfr. ESUs, see also Box 1). We demonstrate that the local biogeographic properties of a population are much more important determinants of effective population size than*

*range size, a commonly used indicator of population size (IUCN). For example, populations of endemic species can achieve levels of genetic diversity that exceed population genetic diversity in more widespread species (Fig 4B and 4C), indicating that effective population sizes are in many circumstances uncoupled from species' geographic ranges.*"

**New L447-452 (Conclusion)**: "*Our findings have major conservation implications, and raise questions regarding the efficacy of formulating management solely based on genetic diversity measured at the species level or range-wide measures of population size. Instead, our results encourage the development a conservation framework based on genetic diversity measured at the population scale and accounting for biogeographic context and life history traits.*"

**New references**:

Mace, G.M., Barrett, M., Burgess, N.D., Cornell, S.E., Freeman, R., Grooten, M., *et al.* (2018). Aiming higher to bend the curve of biodiversity loss. *Nat. Sustain*

Jetz, W., McGeoch, M.A., Guralnick, R., Ferrier, S., Beck, J., Costello, M.J., *et al.* (2019). Essential biodiversity variables for mapping and monitoring species populations. *Nat. Ecol. Evol.*

**Reviewer #3**

**21.** *In this manuscript, the authors conduct a meta-analysis of local genetic diversity, attempting to untangle a large number of hypothesized effects on it. A set of very complex analyses is undertaken, resulting in models that are almost certainly seriously over-fitted. The vast majority of the variance in these models is accounted for by* ***the random effects****, which* ***may control out signal****. The tiny effect sizes of the fixed effects are then presented as important conceptual advances in our knowledge. I expand on these criticisms below. I do think it is worth doing a meta-analysis of local genetic diversity, so I have no problem with the core concept of the paper. Whether it is as 'crucial' (etc) as the authors repeatedly claim in the manuscript is more debatable.* ***There seems to be a lot of hyperbole that is best deleted****; the much more neutral but informative text in Box 1 is far more compelling, in my opinion.*

**Reply**: We thank the referee for these comments. We agree that the definition of the random structure and the fixed structure of mixed models is decisive for finding accurate and robust signals (e.g. through preventing pseudoreplication and multicollinearity). We now fully exploited these issues while re-formulating and re-modeling some of our hypotheses, resulting in considerable increases in variance explained by our fixed variables (from 0.09 to 0.32 for the animal kingdom model, and from 0.05 to 0.10 for the plant kingdom model). The remaining part of the variance most likely is due to a complex mixture of methodological, phylogenetic and functional trait effects as inevitable contributors to $GD_P$. Briefly, we demonstrate that the conclusions from our original manuscript hold, but that we were able to explain additional variance through (i) accounting for modeling issues, (ii) removing redundant variables, and (iii) replacing some of these redundant variables by novel variables with strong hypotheses (e.g. effect plant longevity and past climate). We simultaneously **toned down** some statements where appropriate. We elaborate further on these issues, and in particular on overfitting and explained variance, in our feedback to the more specific comments below.

**22.** *I also suggest that factors that promote local genetic diversity (what they label GD_P) may not necessarily promote beta genetic diversity (**perhaps even a negative relationship between local and beta?**), such that the overall implications of the findings for conservation are more limited than the authors imply. Overall, as suggested in L89, we know quite a lot about species-level genetic diversity, and although the authors do supply some valid reasons why it is **important to also know about population genetic diversity, I am not convinced that it is as important as they claim**.*

**Reply**: We agree, and in fact we already suggested that positive effects on $GD_P$ may translate into negative effects on $GD_S$ in the previous version of the manuscript (Line 253-255). Because we understand the importance of this issue, we made the distinction between $GD_P$ and $GD_S$ more clear in the new version of our manuscript (please see **comment 7**), through considerably elaborating on the difference between $GD_P$ and $GD_S$ in the Box (Box 1) and through feeding back to this issue in the discussion.

**New L378-389:** "*It has been demonstrated that regional genetic diversity at the species level (Box 1) decreases toward the poles in amphibians, mammals and fish*[45,49,52]*; an effect attributed to temperature-dependent mutation and diversification rates. Our results illustrate that population genetic diversity ($GD_P$) and genetic diversity at the species level ($GD_S$) can have strongly different spatial patterns, likely as a result of the interplay between biogeography, climate and species traits together shaping local effective population size. This result has strong implications for the management of local populations with distinct evolutionary histories (cfr. ESUs, see also Box 1). We demonstrate that the local biogeographic properties of a population are much more important determinants of effective population size than range size, a commonly used indicator of population size (IUCN). For example, populations of endemic species can achieve levels of genetic diversity that exceed population genetic diversity in more widespread species (Fig 4B and 4C), indicating that effective population sizes are in many circumstances uncoupled from species' geographic ranges.*"

**L837-859 (New Box 1)**. *Population genetic diversity ($GD_P$) vs. intraspecific genetic diversity ($GD_S$)*

*All differences between $GD_P$ (local scale) and $GD_S$ (range-wide scale) can be reduced to the sampling scale, i.e. the scale at which individuals were sampled to calculate GD. Specifically, $GD_S$ does not capture the effects of the local environmental and biogeographic context on individual populations because the sampled individuals originate from distinct populations that evolved independently. $GD_P$, on the other hand, is calculated for a population of individuals sharing the same eco-evolutionary history. Therefore, $GD_P$ is commonly used in population genetic studies as it captures the effects of the local environment and more contemporary processes (mutation, gene flow, drift and selection), as well as genetic signatures of events in the more distant past. Distinct local populations, each featured by a particular $GD_P$ value, are also referred to as evolutionary significant units (ESUs), i.e. distinct population units that require separate management because they experienced independent evolutionary histories*[8].

*While similar eco-evolutionary and biological processes may drive both $GD_P$ and $GD_S$, the outcome of these processes on $GD_P$ and $GD_S$ can be very different. For example, at the periphery of a species distribution, $GD_P$ is expected to be low due to low connectivity to other populations and thus reduced gene flow. The same biogeographic context (i.e. at the distribution edge), however, may increase $GD_S$ across individuals sampled in independent edge populations because these populations have distinct genetic signatures.*

*Most species are facing population declines on local and regional scales, and can benefit considerably from local conservation efforts that can prevent considerably population extinctions. Therefore, a $GD_P$ baseline (which defines how genetic diversity is predicted to vary according to the environmental and biogeographic properties of populations within and across species) to which conservation practitioners may tailor their conservation strategy, could facilitate local population restoration before extinctions and, eventually, major range contractions occur.*

**23.** ***Overfitting****. The modelling is a major issue in my opinion. The authors fitted hugely complex models (see Models 1, 2 and 3 in the Methods section), with 'all possible variants of the full model' (L388) and then selected the one with the lowest AICc. The only other models considered as alternatives were ones with delta AICc less than 2. AIC does not solve problems associated with* ***multicollinearity or spatial autocorrelation****, and for these reasons among others, the modelling approach taken in this manuscript strikes me as being as close to guaranteeing over-fitting as one can get. (I find it ironic that the authors say that this approach is 'for the sake of simplicity', L389!) I am almost certain that the models contain considerable artefact. Various of the diagnostics reinforce this impression for me, including the strong kurtosis of the residuals and the near-zero error variance. To exemplify, this is the 'best' model (i.e. after simplification) for the taxonomic modelling, according to Supp Table 4: 'Best_model = lmer(GDp ~ |Latitude| + Altitude + Position + Temperature + Precipitation + Humidity + Phylum*Temperature + Phylum*Precipitation + Phylum*Humidity + Phylum*Altitude + Phylum + (1 | Genus/Species) + (1 | marker/loci) + (1 | SampleSize), data = datsc, REML = FALSE)' Tiny effect sizes. Within all that complexity, the overwhelming majority (more than 90%) of the explanatory power was in the random effects (L131), leaving very small effect sizes (totalling 9%) for the fixed effects, which are the core results of the manuscript. The explanatory power of the strongest fixed effects that is then reported in the next few lines (e.g. 32% for phylum, 25% for phylum:latitude interaction) is misleading because these are percentages of 9%, actually meaning 3% and 2% of the overall variation respectively, in these two cases. Those examples are from the taxonomic analysis; the equivalent numbers for the other two analyses are nearly identical. Fig 2 is thus also highly misleading.* ***Could the low explanatory power of the fixed effects be to do with always including 'genus/species' in the random effects?*** *If I have understood this right, this may a* ***priori remove most meaningful signal from life history variables like dispersal type and pollen vector type, and probably other fixed effects too. (****Note: L197 seems to reinforce my suspicion that this is a problem in the analysis.) This probably seriously distorted the main findings of the manuscript.*

**Reply**: It was/is a true challenge finding the right balance between model completeness and model complexity. Population genetic diversity is expected to be shaped by a complex interplay between spatial, biogeographic, biological and environmental variables. Not considering these aspects simultaneously may thus render an incomplete or biased representation of the drivers of population genetic diversity across space and taxa. Overly complex models, on the other hand, also risk biased findings. We took the following actions:

**To avoid collinearity**, we already performed a PCA on all climatic variables, and now we did the same for the three *animal* life history traits (Size, Longevity and Fecundity), rendering two independent life history covariates (PC_SizeLongevity and PC_Fecundity). The plant life history traits

showed no signs of collinearity (see MCA factor map below, obtained through a multiple correspondence analysis, MCA). We thus included the original plant life history traits in our models. We also stress that correlations were low (<0.3) for most pairwise variable comparisons and always <0.7 (see correlogram below and Supplementary Methods).

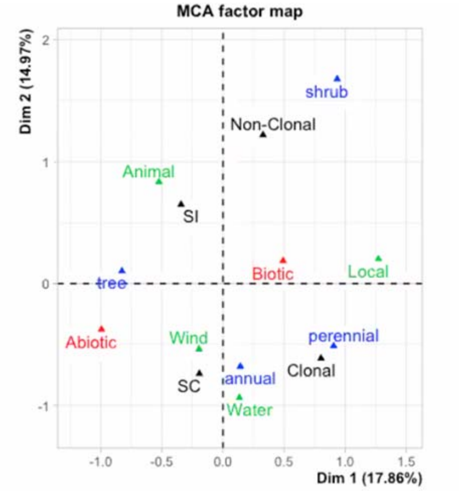


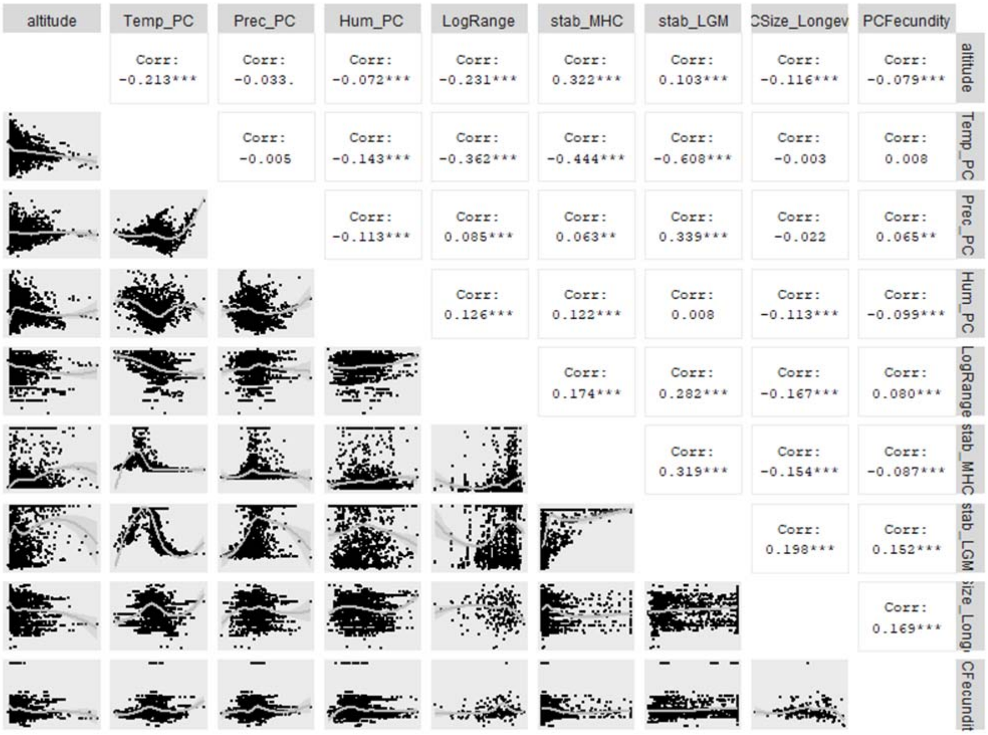


To **eliminate pseudoreplication** caused by taxonomic and methodological issues, we are bound to include "species" as a random effect (please also see comment **3** where we tested "study" as an alternative random factor, but this increased model complexity and deceased $R^2_m$). Including "species" as a random factor does absorb a little variance explained by the life history traits, but it predominantly reflects random processes (relatedness and methodological noise) not captured by life history traits. We tested this by running a model with and without "species" as random factor and with only the life history traits as fixed effects. Including "species" slightly decreased $R^2$m in the animal and plant kingdom model (from 0.16 to 0.12 and from 0.10 to 0.07, resp.), but it hugely

increased $R^2c$ in both kingdom models (from 0.21 to 0.65 and from 0.12 to 0.78, resp.). Model quality also increased substantially when including "species" (AIC decreased from -1855 to -2539 in animals and from -2627 to -4045 in plants). We should not ignore this random structure to prevent misinterpretation of fixed effects.

To nevertheless **simplify the random model structure**, we removed the "genus" effect, which had a very low contribution, as well as the "loci" effect. Instead, we weighted model residuals based on number of loci and sample sizes through a frequently used weighing factor [1/sqrt((log(Loci*SampleSize)))] (see also comment **15**).

To further lower model complexity, we now removed "Latitude" and "Longitude" from all models (based on **comment 17 from reviewer #2**). Instead**,** we tested for **spatial autocorrelation** on model residuals using autocorelograms (see **New lines** below). This shows that our models properly account for potential autocorrelation (no spatial autocorrelation was found in the model residuals), justifying the removal of latitude and longitude from our models.

Note that with all these changes, our **conclusions remained very similar** (e.g. plants have significantly lower $GD_P$ than animals, and the effect of life history traits on $GD_P$ depends on biogeography) while the amount of **variance explained by our fixed variables increased from 0.09 to 0.32 for the animal kingdom model, and from 0.05 to 0.10 for the plant kingdom model**. Finally, we would like to stress that our intention of rescaling $R^2m$ was not to mislead the readers of our manuscript, but rather to remove random variance from the results. As suggested, however, we now avoided rescaling the contributions to $R^2m$. Instead, to visualize fixed variable importance, we took an AIC-based approach considering all models below $\Delta AIC < 4$ and quantifying the relative importance of each term to identify the most important predictors of animal and plant $GD_p$ respectively (see new Fig. 2).

**New L525-533 (Methods):** "*In all models, we (i) weighted model residuals by the number of markers and sample sizes through a frequently used weighing factor in meta-regressions*[110] *and allowing to take into account the precision of the estimate (more weight is given to estimates with a higher precision, i.e., with higher sample size and number of loci)*

$$1/\sqrt{log(Loci \times SampleSize)}$$

*, (ii) included "**Marker**" (co-dominant markers vs. dominant markers vs. enzymes) to control for non-independence within marker types, and (iii) incorporated "**Species**" (extracted using the R package Taxize) to control for non-independence due to both phylogenetic relatedness and study-specific methodological aspects such as $H_E$ estimation methods and sampling protocols."*

**New L511-514**: "*To reduce collinearity among these life history traits, data were synthetized into two principal components, the first one (hereafter "**PC_SizeLongevity**") being positively associated with Size and Longevity and the second one (hereafter "**PC_Fecundity**" being positively associated with Fecundity (Supplementary Methods*)."

**New L572-579**: "*To test for spatial autocorrelation, we used an autocorrelogram approach assessing the relationship between Moran's I of model residuals and pairwise geographical distance*[110]*. We did not find evidence for patterns of spatial autocorrelation neither for the animal kingdom model nor for the plant kingdom model (Fig. S4, Moran'I was weak whatever the class of distance), indicating that spatial autocorrelation is unlikely to impact estimate inferences of these models*[111]*. We therefore did not include spatial terms in the animal and plant models*[111]*. Interestingly, we perform the same*

*procedure on the raw $GD_P$ data (Fig. S4) and we only identified spatial autocorrelation at a very fine spatial scale for animals and to a lesser extent for plants*."

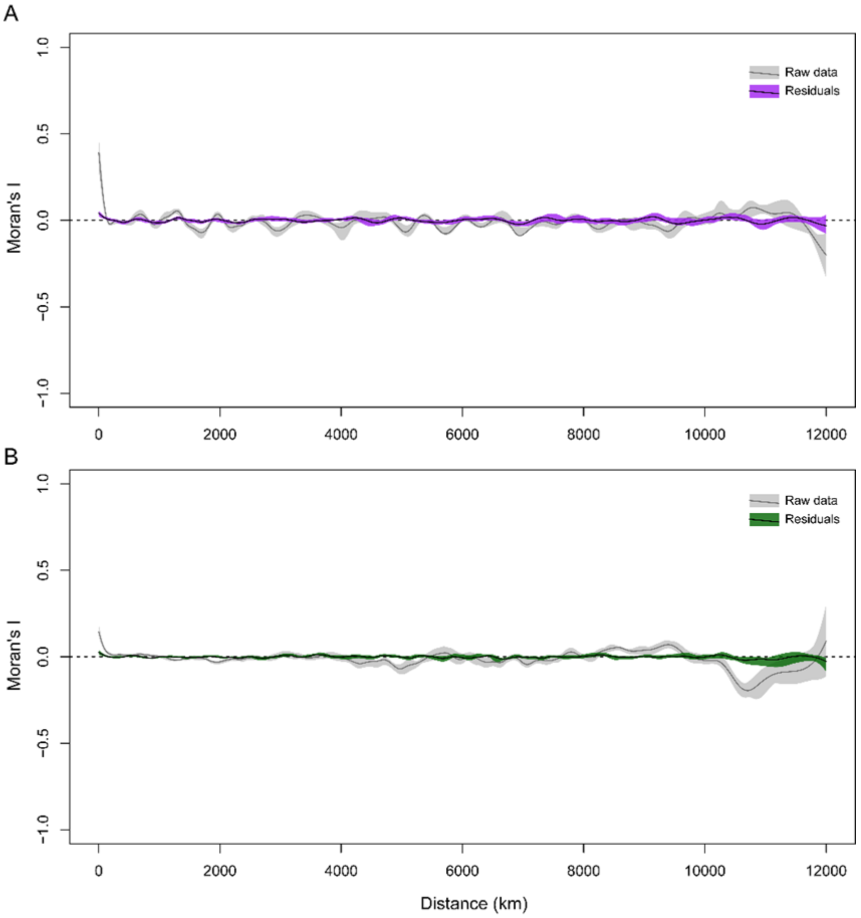


**Fig. S4.** Autocorrelogram of Moran's I across pairwise population distances for animals (upper panel) and plants (lower panel) on raw data and model residuals. The near-zero spatial autocorrelation on model residuals across space demonstrates that our model adequately accounts for spatial autocorrelation.

**New L159-168 (Results)**: "*To explain spatial and taxonomic variation observed in $GD_P$, an exhaustive model was generated for each kingdom separately to assess potential effects of biogeography (i.e. the position of each population relative to the core and edges of the species' range), life history traits (longevity, body size and fecundity reduced to two principal components, and species' range and altitude as a proxies for dispersal ability and niche width, see Methods), contemporary climate (temperature, precipitation, humidity and altitude) and long-term temperature stability during the Last Glacial Maximum (LGM), and during the mid-Holocene (MH) on $GD_P$. Together, these fixed effects explained 32.1% (animals) and 10.2% (plants) of global $GD_P$ patterns. Models' residuals did not display signs of spatial autocorrelation, whereas spatial autocorrelation was detected at a very fine spatial scale for the raw $GD_P$ data especially for animals (Fig. S4)*.**"**

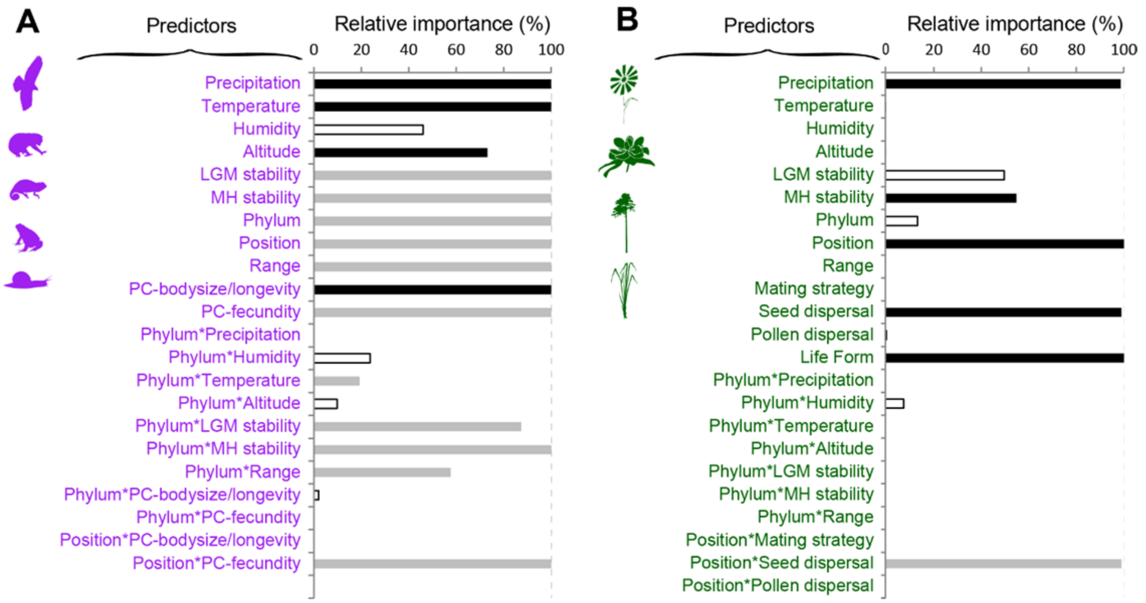


**Fig. 3. Relative importance of predictors used in the animal (A) and plant (B) explicative models respectively**. An information theoretic approach was used to identify the most important predictors of animal and plant $GD_p$ respectively. Predictors with relative importance higher than 50% (i.e. retained in at least half of the best models with ΔAIC of 4 or less) are considered as significant contributors of $GD_p$. Color code indicates "non-significant" predictors (RI<50%, white bars), "significant" predictors not depending on phylum or on the relative position of the population (RI>50%, black bars), and "significant" predictors depending either on phylum or on the relative position of the population (RI>50%, grey bars). When bars are absent the relative importance of the predictor is 0%. Please see Tables S8 and S9 for all models < ΔAIC of 4.

**24.** *Here is another aspect of the analysis that does not fill me with confidence. From L353: 'we calculated orthogonal polynomials from the latitudes using the function 'poly' from the R package Stats, rendering two statistically independent latitudinal variables: the first one (hereafter "Latitude") was correlated with the original latitudinal data, and the second one (hereafter "|Latitude|") with the distance to the Equator. While "Latitude" and "Longitude" were included to account for spatial autocorrelation, "|Latitude|" accounted for variation in ecological and environmental conditions, especially through its association with temperature, photoperiod and historical range expansions.' If I have understood correctly, the absolute latitude variable is simply the square of the latitude. Given that latitude has both negative (southern hemisphere) and positive (N. hemis) values, while the overall linear correlation between the two variables is close to zero, calling the two variables 'statistically independent' is nonsense. This is well illustrated by the top-left graph in the Supp Methods 'Figure', which shows a perfect non-linear relationship.*

__Reply__: In line with this remark, we removed all geographic variables from the models (please see **comments 7** and **23**).

**25**. *The Methods section and Supp Fig 2 implies that the search terms for finding datasets all included only the plural 'populations' as exact terms, and not the singular 'population'. Being within double quotations presumably only found exact matches, thus potentially missing a lot of relevant literature. If this suspicion is true then it is a serious issue for this manuscript.*

**Reply**: Peer reviewed studies assessing the genetic diversity of one population are extremely rare to non-existent, as far as we can assess, so we are very confident that the studies used in our quantitative review constitute a highly representative sample of all studies that published $GD_P$ data. In addition, studies showing population genetic diversity for only one population have negligible value for our quantitative review, because they do not capture any spatial or environmental variation in $GD_P$ among populations.

*There are many problems with the level of explanation and presentation of the manuscript, and I spent much longer than I should have needed to, trying to figure out what was done and what the results are showing. I urge the authors to have more respect for reviewers' time. We should not have to spend ages guessing what things are, trying to find explanations, etc. Here is just a selection of the problems:*

**26**. *The literature search excluded 'pages > 50' (Supp Fig 2) but this is not explained.*

**Reply**: We now clarified this in the caption of Supp Fig 2: "Where google scholar records rendered more than 50 pages of records ("plant" "populations" and "animal" "populations"), only the first 50 pages were considered."

**27.** *Readability is reduced by use of abbreviations in the text (e.g. Ne, H_E, GD_P, GD_S).*

**Reply**: We now removed abbreviations Ne and HE from the text.

**28.** *There are some problems with the referencing (a few author-date, not numbered; some of the numbering is wrong). E.g. L65, L92.*

**Reply**: We carefully checked the referencing and corrected where required.

**29.** *L166 refers to a Fig65B!*

**Reply**: We apologies for this oversight. We corrected accordingly (Now Fig. 5C).

**30.** *In Fig 1b I cannot tell which phylum is which, nor what the units are on the axes.*

**Reply**: We now added the respective phyla to the figure (now Fig. S2). Units correspond to units on the main panel. This was added to the caption. Please see **comment 12** for an updated version of the respective figure.

**31**. *Fig.2 caption mentions red and green colours - bad practice for inclusivity! I actually could not see any colours in this figure until I zoomed in to 350%, and even then found it difficult to distinguish them. It was only at 500% that I could distinguish in Fig 4. In these cases it matters little because I know which taxa are plants and which are animals, but Supp Methods funnel plot figure (unlabelled - at the end of the document) is a nightmare, especially for a red-green colour-blind person like me!*

**Reply**: We apologize for causing this inconvenience. We actually did attempt to account for colour-blindness by adding the names of the phyla to the figure, but for absolute clarity we now also changed the shape of the dots, and we switched from red to purple in all figures. Please let us know if these new figures are more readable for colour-blind persons; if not we will figure out another solution for improving it.

**32**. *Supp Fig 1 is very poorly explained.*

**Reply**: Supp Fig 1 is no longer part of the manuscript.

**33.** *The Supp Tables need proper explanation. For example, there are three latitude columns (Latitude, latitude and abs(latitude)) in Supp Table 1; it needs to be explained that one is 'raw', one is linearly rescaled and the other is the 'absolute' latitude variable, which is just the square of latitude (then rescaled, I guess). This Table refers to the supplementary methods for explanation, but the three latitude variables are not explained there. Users should not have to guess these things.*

**Reply**: We removed all latitude variables except for the raw latitude, and provided a meta-data sheet with explanations for each variable (see **Table S3**).

**34.** *Supp Methods residual plots: various problems. For example, Fig A is one graph but its caption says there is a left panel and a right panel. Fig B has 5 panels, not reflected in the Supp Tables. Etc. Again, far too little explanation.*

**Reply**: We now re-generated the residual plots and moved them to the supporting tables, where model outcomes are presented (Tables S4-S7).

Reviewer #1 (Remarks to the Author):

Thank you for revising your manuscript which now makes a nice contribution to the field of population genetics and conservation.

My comments and suggestions were all properly addressed. And I believe that of the other reviewers as well. Please, find below some suggestions for improving how the text reads and to avoid controversies.

The text often refers to evolutionary potential and fitness when talking about GDp and what it indicates. But all data in this analysis come from neutral marker diversity studies, so the link with fitness (and adaptability) is not obvious. This should be mentioned in the text using caution when linking GDp and fitness (this is said line 397, somewhat a bit far in the text). The link with fitness and adaptability is really indirect for such markers, through demographic processes. And, as revealed by this analysis and by previous studies, restricted distribution plants can have high GDp while some largely distributed ones have low GDp, providing little information as to their fitness (there are several examples in the Pinaceae).

Line 57, maybe a precision here: GDp is not the primary indicator used for delineating ESU, it is the number of different lineages range-wide or in a portion of the range, thus a measure of distinctiveness. GDp complements this nicely and is more often used to define management units for the reasons given lines 59 and 60.

Line 117: are all phyla listed of the same taxonomic level. I think not (I see clades and orders at least), please homogenize.

Lines 162-163 “Together, these fixed effects explained 32.1% (animals) and 10.2% (plants) of global GDP patterns”. Perhaps insist in the text that, while this explanatory power is significant, much of the variation remains unexplained, indicating a lot of idiosyncrasy with species and biological types, calling for accumulating data on individual species, for example for conservation purposes.

Lines 225-226 “Specifically, we found that core populations systematically harbour higher genetic diversity than edge populations”. You say immediately after that while true, this relationship is “subtle”, meaning hardly significant I suppose. Thus, it would be logical to indicate that the trend is tenuous when you highlight it in your conclusion. This is important as there is a lively debate in the field of conservation (including among managers) as to whether or not any emphasis should be placed in edge populations (if they have less diversity, why conserve them?).

Lines 309-311: can we not see here an effect of humans (in addition to faster molecular evolution rate in small animals), for example through hunting?

Lines 360-362: the sentence is not clear, please reformulate.
The set of factors mentioned in the conclusion lines 429-430 vs line 436 are repeated, but not completely. Please, homogenize and perhaps avoid repeating.

Sincerely,
Bruno Fady

Reviewer #2 (Remarks to the Author):

The current version of the manuscript have adequately tackled a significant part of the corcerns I had. Now is easier to follow the set of decission, rules and methods behind the study. Authors have also included new analysis, inlcuding those related with the role of climtic stability over time. Those

improvements have also brought a higher predictive ability of the models.

I am however still concern on various details that need of attention.

a) Mainly for plants, the current set of predictors are still only able to explain a small fraction of the total variance. It would be more fair if authors would expand on this idea and mention/discuss, even briefly, what are potential processes not included in their analysis playing a role in the spatial pattern of genetic diversity.

b) Is not clear what is the source for paleoclimatic information. Is it Worldclim?

c) For amphibians and mollusc, climatic stability sinde the mid- Holocene seems to be more important than climatic stability since the LGM. I can not wonder but try to think on the processes and factors, related to climatic stability, that may have influenced genetic diversity since the mid-Holocene but not since the LGM. Authors need to come with a clear presentation of processes explaining the impact of climatic stability since the Holocene in genetic diversity. So far is not clear.

Hope you find these comments useful to improve your study

Reviewer #3 (Remarks to the Author):

I was previously reviewer #3. This manuscript has improved a lot, and many of the responses to criticisms were good. However, I think there are still some quite serious problems:

Although the removal of genus as a random effect has helped and there has been better model checking and treatment of multicollinearity, the models are still very complex and likely overfitted in my opinion. I did not see any attempt to really address this over-fitting issue, for example by prediction to hold-out data (preferably a whole region of the world held out, so as not to give overly optimistic results because of the autocorrelation structure).

The effect sizes are a bit larger, but still small, especially for plants.
Some over-hyping remains.
Problems of inference remain (inferring causation from correlation).
The issue with the sampling of the literature has not been addressed, but instead misunderstood.
Most of these remaining criticisms should be quite self-explanatory, or at least clear from my previous criticisms, and I just expand on the last one below, for clarity, before moving on to some more minor/specific issues.

The sampling of the literature. I am still concerned that by the use of only the plural form ‘populations’ in the searches you may have missed a lot of relevant papers. And I still think this is a serious issue for this manuscript. Unfortunately you seem to have misunderstood my point here. My criticism was: ‘[T]he search terms for finding datasets all included only the plural ‘populations’ as exact terms, and not the singular ‘population’. Being within double quotations presumably only found exact matches, thus potentially missing a lot of relevant literature. If this suspicion is true then it is a serious issue for this manuscript.’
Your response was:
‘Peer reviewed studies assessing the genetic diversity of one population are extremely rare to non-existent, as far as we can assess, so we are very confident that the studies used in our quantitative review constitute a highly representative sample of all studies that published GDP data. In addition, studies showing population genetic diversity for only one population have negligible value for our quantitative review, because they do not capture any spatial or environmental variation in GDP among

populations.'
Thus you considered it not to be an issue and apparently did not even try using the singular form. However, my point was actually that by using the plural form you miss wording such as 'population dynamics', 'population distributions', 'population-level genetic diversity', 'cross-population', 'multi-population' and many other forms of wording that include the word 'population' but not 'populations'. Many of these are effectively plural forms, and all can refer to multiple populations, but they do not have the s on the end of the word 'population'. My concern therefore remains, and it has nothing to do with suggesting that you include single-population studies, but instead that you may miss lots of multi-population studies when you require there to be an s on the end of the word 'population'.

Previously I also said that the authors should respect reviewer time. Although there are good things such as a comprehensive response letter (though not entirely accurate – see Specifics), it is disappointing that the manuscript has again not been properly checked. To take one example, L552-3: '(26 models for animals and 119 models for plants, see Tables S9 and S10)'
There is no Table S10. The authors appear instead to be referring to Tables S8 and S9. If so, then the figure of 119 models does not match the 116 models shown in Table S9.

SPECIFICS

You said in your response letter "we switched from red to purple in all figures" – err, no you did not. Fig 1B has a red-green colour ramp! It is really not helpful. I can see some patterns but have no idea whether I can see all of them. This must change.
Similarly, Fig.S3 might have different symbols but they are small and the symbols are many, so the colours matter. And they are red vs green. Not helpful and also inconsistent with the colour scheme in the main paper.

In the response letter you said 'we removed all geographic variables from the models', but absolute latitude appears in the 'kingdom model' (Table S4) and the 'phylum model' (Table S5), and Fig. 2B, and the first paragraph of the Results, along with Fig.1, refer to analyses involving latitude.

The core-periphery results start off by being corroboration of a hypothesis, and then a 'general, phylum-independent pattern' that 'is relatively subtle' (L227), but then this pattern suddenly and inexplicably becomes a 'law' in L431 (and in the Abstract), which is surely going too far!

I cannot easily find a data sources reference list.

**REVIEWER COMMENTS**

**Reviewer #1**:

Thank you for revising your manuscript which now makes a nice contribution to the field of population genetics and conservation.

My comments and suggestions were all properly addressed. And I believe that of the other reviewers as well. Please, find below some suggestions for improving how the text reads and to avoid controversies.

**1.** The text often refers to evolutionary potential and fitness when talking about GDp and what it indicates. But all data in this analysis come from neutral marker diversity studies, so the link with fitness (and adaptability) is not obvious. This should be mentioned in the text using caution when linking GDp and fitness (this is said line 397, somewhat a bit far in the text). The link with fitness and adaptability is really indirect for such markers, through demographic processes. And, as revealed by this analysis and by previous studies, restricted distribution plants can have high GDp while some largely distributed ones have low GDp, providing little information as to their fitness (there are several examples in the Pinaceae).

**Response**: We thank the referee for this suggestion. We agree that showing more caution with respect to the putative fitness implications of GDp can help avoiding controversies. Starting from the abstract and the first paragraph of the introduction, we have nuanced the link between GDp on the one hand, and fitness and adaptive evolution on the other hand. We now clarify that this link can only be indirect, as a consequence of demographic processes (leading to inbreeding) and hitchhiking, and we now refer to "evolution" in general, rather than to *adaptive* evolution where appropriate.

**Abstract: New L30-32**: "*Specifically, through its relation with inbreeding and demography, genetic diversity measured in local populations ($GD_P$) is assumed representative for population fitness and eco-evolutionary dynamics.*"

**Introduction: New L55-57**: "*Through its relation with inbreeding and demographic processes*[6,7]*, population genetic diversity ($GD_P$) is often used to support and complement the identification of "evolutionary significant units" (ESUs, Box 1),…..".*

**Introduction: New L102-104**: "*Moreover, while neutral $GD_P$ partially reflects the demographic population history ~~shaped by past adaptive processes~~, part of $GD_P$ arose through hitchhiking with adaptive genetic variants.*"

**Discussion**: **New L441-442**: "*…evolution and adaptability of species.*" → "*…evolutionary dynamics and long-term sustainability of populations.*"

**2**. Line 57, maybe a precision here: GDp is not the primary indicator used for delineating ESU, it is the number of different lineages range-wide or in a portion of the range, thus a measure of distinctiveness. GDp complements this nicely and is more often used to define management units for the reasons given lines 59 and 60.

**Response**: We appreciate this comment and now rephrased this sentence.

**Introduction: New L55-57**: "*Through its relation with inbreeding and demographic processes*[6,7]*, population genetic diversity ($GD_P$) is often used to support and complement the identification of "evolutionary significant units" (ESUs, Box 1),…..".*

**3.** Line 117: are all phyla listed of the same taxonomic level. I think not (I see clades and orders at least), please homogenize.

**Response**: Two different taxonomic levels were used: classes (most animal phyla) and broader "clades" (the plant phyla and the molluscs). We could not homogenize the taxonomic level because other divisions are

typically used in plants than in animals (e.g. there is no consensus about the "class" rank in the plant kingdom), and because sample sizes would become too small (e.g. mollusc classes). We thus preferred a division into well-defined and well-known groups that were comparable in sample size (although the molluscs remain a small group). We clarified the taxonomic level of the "Pines" group, which refers to the respective clade (Pinopsida) rather than the order (Pinales): **L115**.

**4.** Lines 162-163 "Together, these fixed effects explained 32.1% (animals) and 10.2% (plants) of global $GD_P$ patterns". Perhaps insist in the text that, while this explanatory power is significant, much of the variation remains unexplained, indicating a lot of idiosyncrasy with species and biological types, calling for accumulating data on individual species, for example for conservation purposes.

**Response**: We agree and added this information to our paragraph dealing with this unexplained variance.

**Discussion L277-279**: "*In parallel, the high species-specificity of $GD_P$ emphasizes the importance of accumulating more data on individual species for further understanding which taxonomic $GD_P$ patterns are key to conservation planning*"

**5.** Lines 225-226 "Specifically, we found that core populations systematically harbour higher genetic diversity than edge populations". You say immediately after that while true, this relationship is "subtle", meaning hardly significant I suppose. Thus, it would be logical to indicate that the trend is tenuous when you highlight it in your conclusion. This is important as there is a lively debate in the field of conservation (including among managers) as to whether or not any emphasis should be placed in edge populations (if they have less diversity, why conserve them?).

**Response:** We did not look at our results from this perspective, but agree that a focus on edge population management is interesting. We thus nuanced the generality of the core-periphery hypothesis and put more emphasis on how the $GD_P$ of edge populations, can actually be similar to that of core populations, depending on species characteristics. This implicates that edge populations should be carefully considered in conservation management.

**Discussion L260-267**: "*Our finding that core populations frequently, but not systematically display higher genetic diversity than edge populations is of major importance as (i) it demonstrates that edge populations can represent non-negligible sources of intraspecific diversity (mostly in plant species, Fig. 5C), and (ii) it emphasizes the discrepancy between $GD_P$ and $GD_S$, with $GD_P$ being sensitive to the biogeographic context of the population. Conservation-wise, edge populations should be considered in light of the species' life-history traits, or genetically compared to core populations to avoid potential loss of unique genetic variation by prioritizing core populations in conservation planning*."

**6.** Lines 309-311: can we not see here an effect of humans (in addition to faster molecular evolution rate in small animals), for example through hunting?

**Response:** Possibly. We now discussed this as an additional explanation for the relatively low $GD_P$ in large animals.

**Discussion L318-320**: "Likely, large animal species also have experienced increased anthropogenic pressures, for example through hunting and fragmentation of their large population territories[74]"

**New reference**: Cardillo, M. *et al.* Multiple causes of high extinction risk in large mammal species. *Science* **309**, 1239–1241 (2005)

**7.** Lines 360-362: the sentence is not clear, please reformulate.
The set of factors mentioned in the conclusion lines 429-430 vs line 436 are repeated, but not completely. Please, homogenize and perhaps avoid repeating.

**Response:** A word was missing from the sentence, rendering it grammatically incorrect and confusing. This is now adjusted (**L372-373**: "…*driven* *by* *species' life history traits,* *by* *the location of a population relative to the species' distribution range, and* *by* *contemporary climate*."). We also removed original lines 429-430 because they repeated fragments from other parts of the conclusion.

Sincerely,
Bruno Fady

**Reviewer #2**:

The current version of the manuscript have adequately tackled a significant part of the corcerns I had. Now is easier to follow the set of decission, rules and methods behind the study. Authors have also included new analysis, inlcuding those related with the role of climtic stability over time. Those improvements have also brought a higher predictive ability of the models.

I am however still concern on various details that need of attention.

**8.** Mainly for plants, the current set of predictors are still only able to explain a small fraction of the total variance. It would be more fair if authors would expand on this idea and mention/discuss, even briefly, what are potential processes not included in their analysis playing a role in the spatial pattern of genetic diversity.

**Response**: Especially for plants, we suspect that the lack of fecundity data related to reproductive output is an important reason for the much lower $R^2m$ in the plant kingdom model as compared to the animal kingdom model (in which we did include reproductive success). We did indeed not discuss this in the previous version of our manuscript but have now added this view.

**Discussion L431-436**: "*In plants, seed bank persistence*[103,104] *and fecundity-related traits (e.g. average seed production) may be strong moderators of population genetic diversity. Indeed, the important role of fecundity in the animal kingdom suggests that the low amount of explained variance of plant* $GD_P$ *may be due to the lack of fecundity metrics in our plant models. Seed set is correspondingly considered an important fitness trait*[105]*, with inbreeding frequently resulting in reduced seed set* [106]".

**New references**:

Campbell et al. 2017 Is Plant Fitness Proportional to Seed Set? An Experiment and a Spatial Model. *The American Naturalist* **190**: 818-827.

Angeloni et al. 2011 Meta-analysis on the association of population size and life history with inbreeding depression in plants. *Biological Conservation* **144**: 35-43.

**9.** Is not clear what is the source for paleoclimatic information. Is it Worldclim?

**Response:** That is correct. We mentioned this in the Supporting Methods, but now also added this information to the main text (**L493**).

**10.** For amphibians and mollusc, climatic stability since the mid- Holocene seems to be more important than climatic stability since the LGM. I cannot wonder but try to think on the processes and factors, related to climatic stability, that may have influenced genetic diversity since the mid-Holocene but not since the LGM. Authors need to come with a clear presentation of processes explaining the impact of climatic stability since the Holocene in genetic diversity. So far is not clear.

**Response:** We believe that amphibians and molluscs are particularly sensitive to past episodes of climate change because of their poor dispersal abilities. Slow movement may have prevented these particular groups

of organisms from keeping pace with past climate change, causing rapid population turn-over during episodes of past climate change, thereby erasing earlier signatures of climate stability on $GD_P$ (e.g. between LGM and MH). In fact, the very same mechanism may explain their currently low levels of genetic diversity (groups with the lowest $GD_P$ at this moment; see Fig. 2): ongoing climate change may have stronger impacts on amphibians and molluscs than any other group of species because tracking climate change is particularly challenging for them. We now emphasized this in the text.

**Discussion L291-295**: "*Limited dispersal may also explain why we did not find impacts of earlier climate stability (since LGM) on $GD_P$ in molluscs and amphibians. Specifically, slow movement may prevent these particular groups of organisms from keeping pace with past climate change, causing rapid and range-wide population turn-over, thereby erasing earlier signatures of climate stability on $GD_P$ (e.g. between LGM and MH).*"

Hope you find these comments useful to improve your study

**Reviewer #3**:

I was previously reviewer #3. This manuscript has improved a lot, and many of the responses to criticisms were good. However, I think there are still some quite serious problems:

**11.** Although the removal of genus as a random effect has helped and there has been better model checking and treatment of multicollinearity, the models are still very complex and likely overfitted in my opinion. I did not see any attempt to really address this over-fitting issue, for example by prediction to hold-out data (preferably a whole region of the world held out, so as not to give overly optimistic results because of the autocorrelation structure).

**Response:** Thank you for this comment. Overfitting can in our case affect the estimates of model parameters, which may alter our conclusions. In order to more explicitly meet this concern, we have now performed two model sensitivity analyses to test the robustness of our models. As the referee proposed, we first excluded, one at a time, large regions from the animal and plant kingdom models respectively. Note that removing large regions often implied removing many data points and thus increasing over-fitting. We therefore limited the size of the excluded regions to allow a maximum data exclusion of 15%. The table below shows the chi-square statistics with p-values of the four most important variables (highest test Chi squares) together with $R^2m/R^2c$ of the animal and plant kingdom models ran on the full (original) dataset as well as on each of six regionally reduced datasets (see **Figure 1** below for the excluded regions). Our model results did not change when excluding regions: the four most important variables maintain their importance in each of the subsets and explained variance estimates only slightly fluctuate around the original estimates.

**Table** showing model output for data subsets excluding parts of the world as depicted in **Figure 1** below. For each subset, the four most contributing variables (highest Chi square) are shown together with their significance (* for p<0.05, ** for p<0.01 and *** for p<0.001). Please see supporting information for review only for full ANOVA tables.

| **Animals** | N excluded | $R^2m/R^2c$ | Position | PhylumxMH | PositionxFecundity | RangexPosition |
|---|---|---|---|---|---|---|
| original model | | 0.32/0.71 | 76.85*** | 51.56*** | 37.51*** | 30.54*** |
| Excl Europe | 345 (13.53%) | 0.32/0.74 | 81.54*** | 41.72*** | 25.64*** | 23.35*** |
| Excl N America | 184 (7.22%) | 0.30/0.71 | 68.31*** | 35.11*** | 34.72*** | 26.36*** |
| Excl S America | 51 (2.00%) | 0.29/0.73 | 80.17*** | 52.49*** | 30.16*** | 29.42*** |
| Excl Asia | 71 (2.78%) | 0.33/0.72 | 82.75*** | 50.39*** | 31.32*** | 30.62*** |
| Excl Australia | 122 (4.78%) | 0.31/0.71 | 69.09*** | 53.16*** | 20.76*** | 24.45*** |
| Excl Africa | 98 (3.84%) | 0.30/0.71 | 74.21*** | 51.71*** | 35.09*** | 26.76*** |

| **Plants** | N excluded | $R^2m/R^2c$ | SeedxPosition | Position | LifeForm | Precipitation |
|---|---|---|---|---|---|---|

| | | | | | | |
|---|---|---|---|---|---|---|
| original model | | 0.10/0.79 | 38.91*** | 34.58*** | 30.41*** | 5.74* |
| Excl Europe | 530 (12.50%) | 0.12/0.78 | 32.10** | 27.35*** | 29.37*** | 6.34* |
| Excl N America | 139 (3.28%) | 0.11/0.79 | 36.70** | 35.31*** | 30.51*** | 5.74* |
| Excl S America | 292 (6.89%) | 0.09/0.80 | 42.20*** | 32.78*** | 25.82*** | 6.25* |
| Excl Asia | 307 (7.24%) | 0.10/0.79 | 47.22*** | 33.05*** | 27.89*** | 4.50* |
| Excl Australia | 108 (2.55%) | 0.10/0.78 | 37.40** | 34.14*** | 26.58*** | 5.84* |
| Excl Africa | 150 (3.54%) | 0.10/0.79 | 38.78* | 31.38*** | 29.11*** | 4.39* |

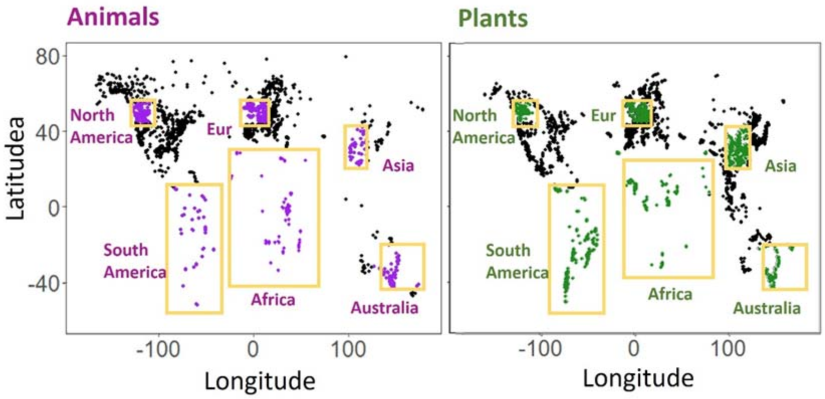


**Figure 1** showing the data excluded for sensitivity testing of model robustness.

Additionally, we performed jackknife procedures to specifically test the robustness of our model parameters when subsequently and randomly leaving out all data points from (i) one squared geographical area of three degrees latitude and longitude, and (ii) one species. As such, this approach deals with the two main concerns of the referee, i.e. whether spatial autocorrelation and random structure compromised model robustness. Please find below the outcomes of our jackknife procedure, demonstrating negligible spatial and taxonomic impact on the model parameter coefficients (tiny confidence intervals). According to these tests, we are extremely confident about the parameter estimates of our models, and hence regarding the conclusions drawn from these analyses.

We added the jacknife procedure and its output to our manuscript:

**Methods. New L582-587**: *" To test the extent to which the spatial (geography) and random (species) structure of our model may affect model parameter robustness due to potential overfitting, we consecutively left out all data points from (i) each random species (219 in animals and 306 in plants) or (ii) each occupied squared geographical area (3 degrees latitude and longitude; 57 areas in animals and 75 areas in plants) using a jacknife procedure, to obtain 95% confidence intervals about each parameter. Our models appear highly robust to both geographic and taxonomic variations (Fig. S5).”*

**New Fig. S5**: “*Model parameter robustness after leaving out random species and geographical areas from plant (upper panels) and animal (lower panels) kingdom model*.”

**Plant kingdom model**: Impact of random effect “species” (upper) and spatial autocorrelation (lower) on parameter robustness

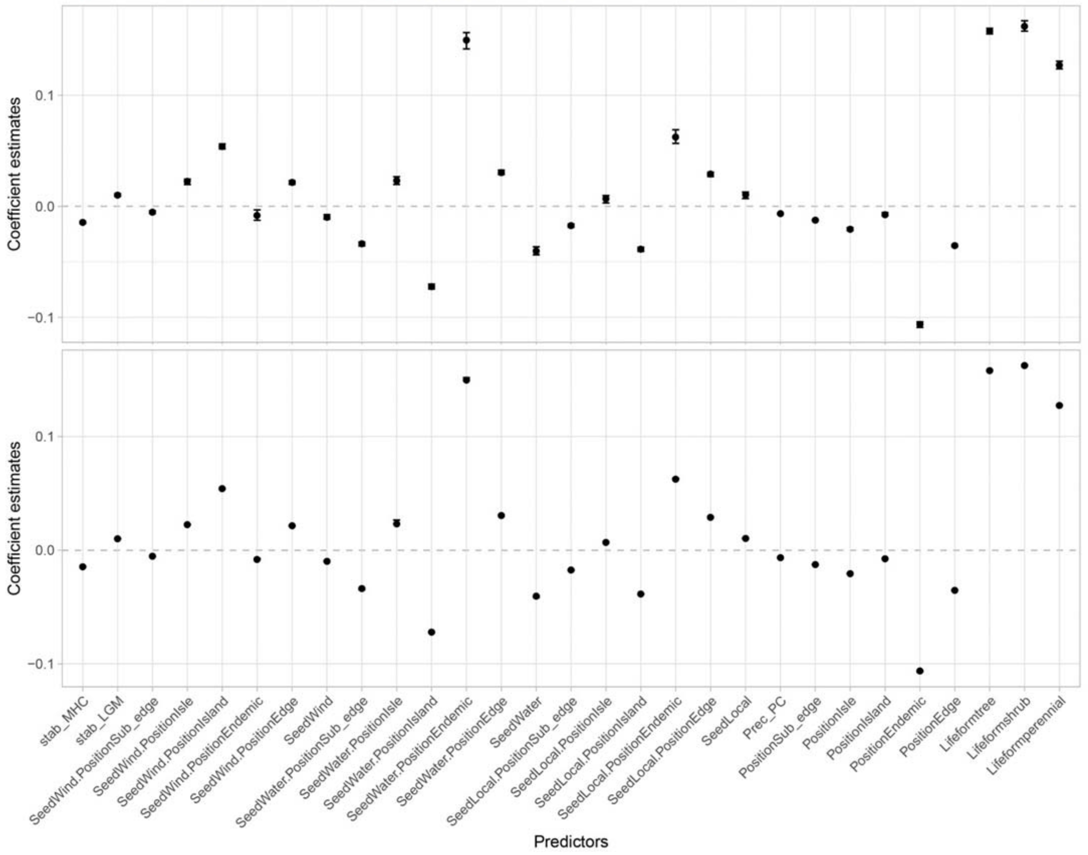


**Animal kingdom model**: Impact of random effect “species” (upper) and spatial autocorrelation (lower) on parameter robustness

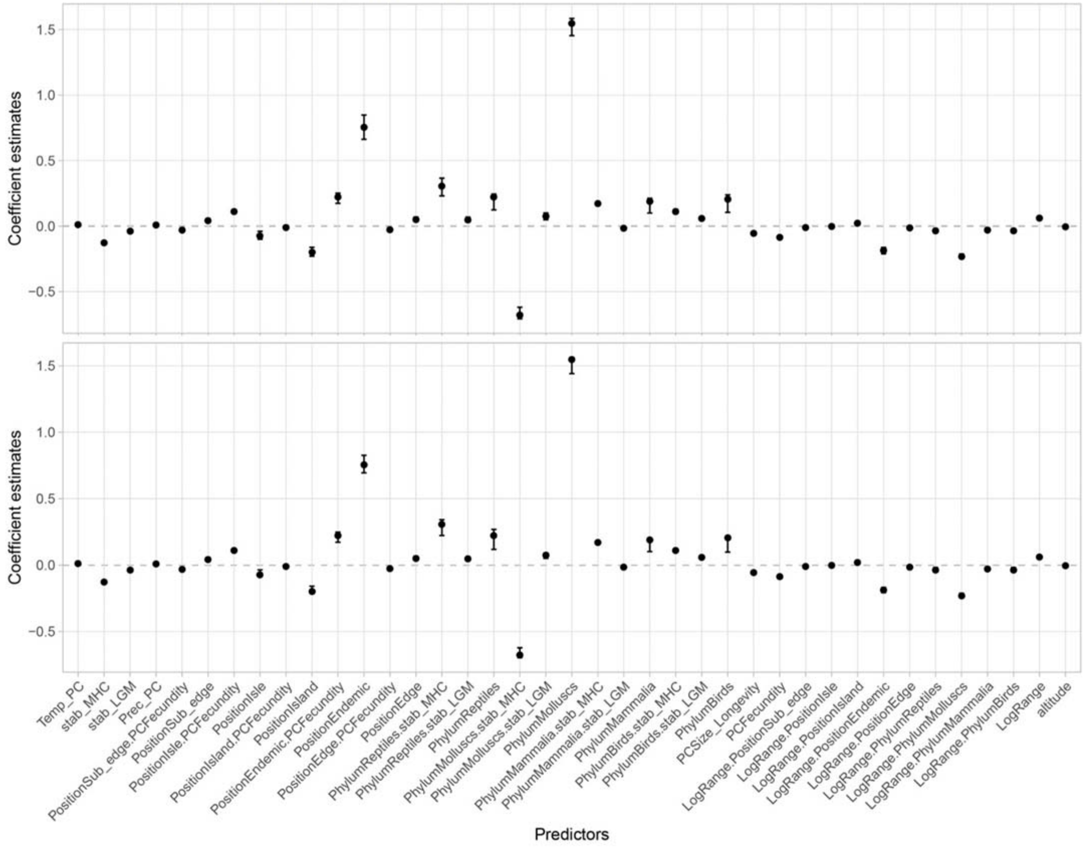

**12**. The effect sizes are a bit larger, but still small, especially for plants.
Some over-hyping remains. Problems of inference remain (inferring causation from correlation).

**Response:** Actually, the effect sizes tripled for animals ($R^2_m$ from 0.09 to 0.32) and doubled for plants (from 0.05 to 0.10) after re-construction of our original models. We truly believe that finding such effect sizes (even though we agree still relatively small for plants) for a the highly dynamic and context-dependent nature of estimates such as expected heterozygosity is not evident. We are therefore convinced that our findings are highly relevant to conservation ecologists and population geneticists. Moreover, the relatively low explained variance in our plant kingdom model opens novel research avenues, as discussed in our manuscript. As proposed by referee 2, please note that we now have added an explanation for the relatively low $R^2$m in plants (please see **comment 8**), and –as requested- we tackled over-hyping in **comment 17** below.

**13**. The issue with the sampling of the literature has not been addressed, but instead misunderstood.
Most of these remaining criticisms should be quite self-explanatory, or at least clear from my previous criticisms, and I just expand on the last one below, for clarity, before moving on to some more minor/specific issues. The sampling of the literature. I am still concerned that by the use of only the plural form 'populations' in the searches you may have missed a lot of relevant papers. And I still think this is a serious issue for this manuscript. Unfortunately you seem to have misunderstood my point here. My criticism was:
'[T]he search terms for finding datasets all included only the plural 'populations' as exact terms, and not the singular 'population'. Being within double quotations presumably only found exact matches, thus potentially missing a lot of relevant literature. If this suspicion is true then it is a serious issue for this manuscript.'
Your response was: 'Peer reviewed studies assessing the genetic diversity of one population are extremely rare to non-existent, as far as we can assess, so we are very confident that the studies used in our quantitative review constitute a highly representative sample of all studies that published GDP data. In addition, studies showing population genetic diversity for only one population have negligible value for our quantitative review, because they do not capture any spatial or environmental variation in GDP among populations.'
Thus you considered it not to be an issue and apparently did not even try using the singular form. However, my point was actually that by using the plural form you miss wording such as 'population dynamics', 'population distributions', 'population-level genetic diversity', 'cross-population', 'multi-population' and many other forms of wording that include the word 'population' but not 'populations'. Many of these are effectively plural forms, and all can refer to multiple populations, but they do not have the s on the end of the word 'population'. My concern therefore remains, and it has nothing to do with suggesting that you include single-population studies, but instead that you may miss lots of multi-population studies when you require there to be an s on the end of the word 'population'.

**Response:** We had misunderstood this comment indeed**.** We now understand that the issue is whether multi-population studies of genetic diversity exist that do not include the plural word 'population**s**'. To assess this issue, we screened 100 randomly selected studies retrieved by using the search terms "expected heterozygosity" AND "genetic marker" AND "x" AND "**population**" (with x representing "plant" or "mammal"), instead of our original search terms "expected heterozygosity" AND "genetic marker" AND "x" AND "**populations**". We could not find one multi-population study that did not mention the plural "populations", even though google scholar searches full articles (i.e. searches are not limited to title and abstract) for key words. While we did find some studies that involved only one population, these singe-population studies were filtered from our literature search because capturing spatial variation in genetic diversity within species requires studies with expected heterozygosity for multiple populations. Again, we did not find any multi-population study avoiding the use of the plural word "populations". Consequently, we are very confident that virtually all multi-population studies were captured by our original search term "populations". Finally, comparing the number of records arising from our original search terms (with "populations") to those with "population" indicates that the difference is actually small (see table below for a limited number of taxa). Only for birds, we found a discrepancy, which is due to the dominance of single-population studies in birds, in which we were not interested.

| Taxon (“expected heterozygosity” AND “genetic marker” AND “taxon” and “populations” vs “population”) | Number of hits when using “populations” | Number of hits when using “population” |
|---|---|---|
| **Plant** | 1720 | 1810 |
| **Tree** | 1760 | 1810 |
| **Mammal** | 746 | 758 |
| **Bird** | 474 | 716 |
| **Frog** | 174 | 176 |
| **Lizard** | 165 | 168 |
| **Mollusc** | 158 | 162 |

As an important side note, having all the studies containing $GD_P$ estimates is not mandatory to obtain robust estimates and conclusions (it is almost impossible to ensure that all studies are actually considered). On the contrary, what *is* very important to get reliable estimates (and conclusions) is to get a large and representative sample of $GD_P$ across space and taxa. We are really confident that our dataset is representative of published papers as we did not detect publication bias (see Fig. S3), and as shown above, changing keywords (population vs populations) do not drastically change the output of the search strategy. We added a line to our methods section to make this clear.

**Methods L483**: “*We primarily focused on multi-population studies in order to capture within-species biogeographic variation*.”

**14.** Previously I also said that the authors should respect reviewer time. Although there are good things such as a comprehensive response letter (though not entirely accurate – see Specifics), it is disappointing that the manuscript has again not been properly checked. To take one example, L552-3:
‘(26 models for animals and 119 models for plants, see Tables S9 and S10)’
There is no Table S10. The authors appear instead to be referring to Tables S8 and S9. If so, then the figure of 119 models does not match the 116 models shown in Table S9.

**Response:** We apologize for this sloppiness**.** We again checked all references to figures, tables and supporting files and corrected the error noted by the referee (incl. 116 models instead of 119), as well as an error in Table S5. Thank you for pointing at these mistakes. We have all read again the MS and we hope all mistakes (see highlighted version of the manuscript) have now all been corrected. Thanks for your careful screening of the MS.

SPECIFICS

**15.** You said in your response letter “we switched from red to purple in all figures” – err, no you did not. Fig 1B has a red-green colour ramp! It is really not helpful. I can see some patterns but have no idea whether I can see all of them. This must change. Similarly, Fig.S3 might have different symbols but they are small and the symbols are many, so the colours matter. And they are red vs green. Not helpful and also inconsistent with the colour scheme in the main paper.

**Response:** Indeed, Fig. 1 was a new figure, and we forgot to apply the adjusted colour scheme to this figure. This is now corrected. The same is true for Fig. S3.

**16**. In the response letter you said ‘we removed all geographic variables from the models’, but absolute latitude appears in the ‘kingdom model’ (Table S4) and the ‘phylum model’ (Table S5), and Fig. 2B, and the first paragraph of the Results, along with Fig.1, refer to analyses involving latitude.

**Response:** That is correct. As mentioned in our previous response letter we included absolute latitude as an “integrative environmental” variable rather than as a geographic variable. Indeed, as written in the MS (**L540-541)**; “*Because distance to equator is thought to be an important moderator of genetic diversity through its*

*association with temperature, productivity, long term stability and historical range expansions, we implemented a linear mixed modeling (LMM) approach testing the impacts of |Latitude|*Kingdom while controlling for non-independence within marker types and species (see above)*." We now replaced "latitude" by "absolute latitude" throughout the discussion to further emphasize this.

**17.** The core-periphery results start off by being corroboration of a hypothesis, and then a 'general, phylum-independent pattern' that 'is relatively subtle' (L227), but then this pattern suddenly and inexplicably becomes a 'law' in L431 (and in the Abstract), which is surely going too far!

**Response:** We fully agree that the core-edge findings should have been discussed from another perspective. Indeed, the observation that edge effects depend on species life history traits has important conservation implications. We therefore adjusted our abstract and discussions about the core-edge hypothesis (see also **comment 5** of referee 1).

**Abstract L40-42**: "*Peripheral populations can consequently, and particularly in some plant groups, sustain similar $GD_P$ as core populations, emphasizing their potential conservation value*.."

**Discussion L224-226**: "*Nonetheless, in some species groups, core and edge populations sustain similar levels of genetic diversity, which has important implications for conservation management*."

**Discussion L260-267**: "*Our finding that core populations frequently, but not systematically display higher genetic diversity than edge populations is of major importance as (i) it demonstrates that edge populations can represent non-negligible sources of intraspecific diversity (mostly in plant species, Fig. 5C), and (ii) it emphasizes the discrepancy between $GD_P$ and $GD_S$, with $GD_P$ being sensitive to the biogeographic context of the population. Conservation-wise, edge populations should be considered in light of the species' life-history traits, or genetically compared to core populations to avoid potential loss of unique genetic variation by prioritizing core populations in conservation planning*."

**18**. I cannot easily find a data sources reference list.

**Response:** All data sources were mentioned in the main text. We now, however, added this source information to the metadata sheet in the Supporting Tables (Table S3), to which we refer in **L116**, and now also in **L512**. Note that Table S2 already included the list of studies from which our $GD_P$ and geographical data were extracted.

Reviewer #3 (Remarks to the Author):

Thanks for again taking my comments and criticisms seriously, as with those of the other referees. I think you have adequately (or better) address all the substantive concerns.
Best wishes, Richard Field.
